# Mr. Paget's Report on Microscopical Anatomy and Physiology

**Published:** 1842-07

**Authors:** James Paget

**Affiliations:** Demonstrator of Morbid Anatomy at St. Bartholomew's Hospital


### PART FOURTH.

### Medical Intelligence.

---

### REPORT ON THE RESULTS OBTAINED BY THE USE OF THE MICROSCOPE IN THE STUDY OF HUMAN ANATOMY AND PHYSIOLOGY. Part I. By JAMES PAGET, Demonstrator of Morbid Anatomy at St. Bartholomew's Hospital.

THE design of the present report is to bring together in the briefest possible space the conclusions regarding the structure and the functions of the several tissues of the human body which have been rendered certain or most probable by microscopic investigation. The task will, it is hoped, be deemed worthy of the labour which has been bestowed upon it; for in no department of medical science has there been so great an addition of facts in the last ten years as in minute anatomy; and in none has the access to knowledge been more difficult. The greater part of the original records of microscopic anatomy are scattered through a multitude of monographs, of brief dissertations, and of essays in the foreign journals, to which few can refer; and in our own language there is no work which affords an adequate notion of their contents. In France, Holland, and Italy there is the same defect; and even in Germany it existed till, very recently, the systems of general anatomy of Henle[1] and Bruns[2] appeared.

This First Part of the report relates to the structure of the general component parts of the body in their complete state; in a Second Part, their several schemes of development will be described.

The writer has endeavoured to keep within the strict limits of the office he has assumed; he has not pretended to do more than report what has been already published; but that he might do so accurately and impartially he has been careful to draw his materials from none but their original sources.

Some of the numerous reference-notes may seem superfluous; but they are inserted in the belief that with their aid it will not be difficult for any one who has access to the works quoted to fill up the outline of knowledge which the text affords. By the aid of some, interesting histories of discovery may be traced; by others, facts may be found which, though they now seem unimportant and are, for brevity's sake, omitted, may hereafter become valuable; by means of others again, the description of the structure of particular organs, and the details of the facts which are related in general terms in the text, may be at once referred to.

The fact of the single origin of all the tissues from primary cells suggests that the most natural arrangement of them must be that in which they are placed in a succession corresponding to the degrees in which, in their perfected condition, they severally deviate from the primary form. And although, from the imperfection of the knowledge hitherto attained, such an arrangement cannot yet be certainly and completely established, there are sufficient advantages in even a partial adoption of it to warrant its employment on the present occasion, with only such modifications as the physiological relations of certain parts seem, in some instances, to render more convenient than a strict adherence to system.

[1] Allgemeine Anatomie, von J. Henle—Leipzig, 1841, being the 6th volume of the new German edition of Sömmering's Anatomie. The present part of the Report was nearly completed when this admirable work arrived in England: but the numerous references made to it will sufficiently prove of how much avail it has been for addition and confirmation to what had been written.

[2] Lehrbuch der allgemeinen Anatomie des Menschen, von Victor Bruns.—Braunschweig, 1841. 8vo.

With this preface, and with the aid of the second part of the report, the plan here followed will be easily intelligible.

I. Blood-corpuscles. The discovery of the blood-corpuscles by Malpighi[1] was one of the first-fruits of microscopic study, and since that event few objects have been more solicitously examined. It is now agreed that they are minute, flattened, transparent cells, containing (at least during one period of their existence) round, or oval nuclei, and having incorporated in them all the red colouring matter of the blood.

*a. Form and Size.* They are circular in man and in all mammalia, except the camel tribe in which they are elliptical;[2] and they are elliptical in all other vertebrata, except certain cyclostomes in which they are circular.[3] In all they are flattened and have rounded borders. Whether their surfaces be slightly concave or convex depends on variations in the quantity of their contents which may ensue either within, or after their removal from, the body. In invertebrata they are less numerous but more varied in form; for the most part they are irregular, granular, roundish, nucleated corpuscles.[4]

It is difficult to discern any strict connexion between the various sizes of these bodies and the other parts of the organism of different animals. Among mammalia those of the elephant are the largest;[5] then come those of the capybara and rhinoceros;[6] then those of man, which have an average diameter of about $\frac{1}{3500}$ or $\frac{1}{4000}$ of an inch.[7] In general those of ruminants are smaller than those of other mammalia; and the smallest yet known are those of the little chevrotain and Napu musk-deer, of which the average diameter is less than $\frac{1}{12000}$ of an inch.[8] An examination of the elaborate tables by Mr. Gulliver shows that the size of the corpuscles in mammalia is not unconditionally proportionate to the size of each animal, or according to the nature of its food. Yet there is evidence enough that in each great division of the class, the size of the blood-corpuscles is, with few exceptions, directly proportionate to that of the animal's body; and that, in general, those of omnivora are larger than those of carnivora, and those of the latter larger than those of herbivora; so that if the kind of food and the size of the mammal be known, the size of its blood-corpuscles may be probably estimated.

In birds there is a greater uniformity of size and shape in the blood-corpuscles than in mammalia, and, according to Mr. Gulliver, a nearer relation between

[1] Athanasius Kircher, Malpighi, and John Swammerdam have all received from different writers the honour of this discovery. Whoever will, may satisfy himself of the justice of the award given in the text by consulting A. Kircher, (Scrutinium Phys. Med. Pestis, Lips. 1659, p. 240 and the context); Malpighi, (De Omento, Pinguedine, &c. p. 42;) and his Autobiography in the Opera Posthuma, (pp. 25, 92, in the Fol. Ed.;) and Swammerdam, (History of Insects, first published in 1669, at p. 31, of Hill's edition of 1778.) Lists of the principal writers on the blood-corpuscles may be collected from the General Anatomies of E. H. Weber, Gerber, and Henle.

[2] This remarkable and unexplained exception was discovered by Mandl in the dromedary and alpacha (Comptes Rendus des Seances de l'Acad. des Sc., Dec. 30, 1839), and has been amply confirmed.

[3] R. Wagner, (Lehrbuch der Physiologie, B. i. 153.)

[4] Wagner, Lehrbuch, and Beitrage zur vergleich. Physiologie. But he regards them as only chyle-corpuscles.

[5] Mandl, (Anatomie Microsc. p. 17.) Owen (Contributions to the Comp. Anat. of Blood-discs, Lond. Med. Gazette, Nov. 15, 1839) says most of them are $\frac{1}{4}$ larger than human blood-corpuscles. According to Gulliver (Appendix to Gerber's General Anatomy, p. 42,) the average diameter is $\frac{1}{2473}$ of an inch, which is nearly accordant with Mandl's statement.

[6] Gulliver, *l. c.* *All the sizes mentioned in this part of the report are stated in fractions of an English inch.*

[7] Perhaps the strictest measurements are those of Mr. Bowerbank for Mr. Owen *l. c.* In these the average was $\frac{1}{3687}$, the extremes being $\frac{1}{4545}$ and $\frac{1}{3279}$. Copious lists of the measurements by different observers are given by Köstlin, (Mikroskop. Forschungen, p. 55;) in the Microscopic Journal, vol. i. and in Mandl, *l. c.* p. 10.

[8] Owen, *l. c.* Dec. 20, 1839; and Gulliver, *l. c.* p. 44.

their size and that of the body. They are the smallest of the elliptical blood-corpuscles, those of the camel tribe excepted; they are generally rather less than twice as long as they are broad, measuring about $\frac{1}{2000}$ by $\frac{1}{4000}$ of an inch, and about six times as long as they are thick. In reptiles, the largest and, by comparison, the thinnest, blood-globules yet known occur; and Wagner[1] remarks it as a general rule, which Mr. Owen confirms, that the longer the branchial apparatus persists, the larger are the blood-corpuscles. Thus, in the Proteus they are about $\frac{1}{350}$ of an inch long, in the Syren $\frac{1}{435}$ by $\frac{1}{800}$ [2]; in the batrachian reptiles generally, about $\frac{1}{1000}$ by $\frac{1}{3000}$; and their thickness is not more than one eighth of their length.[3] This rule, however, fails when one comes to fish, in which the branchial apparatus is persistent and perfect; for in them the blood-corpuscles, though resembling those of reptiles, are generally smaller and less elongated.[4]

*b. Structure and Composition.* The blood-corpuscles are generally regarded as primary nucleated cells, and no one doubts that those of birds and the lower vertebrata consist of an external cell, formed of an extremely delicate, soft, and elastic membrane, in and within which all the colouring matter seems to be contained, and of an internal parietal nucleus, generally similar in form to the cell, but about one fourth its size, colourless, and in the large corpuscles of some of the amphibia containing a number of distinct granules.[5]

It is questioned, however, especially by Valentin, Wagner,[6] and Gulliver,[7] whether the corpuscles of mammalia have nuclei, or whether the central spot be not merely produced by the accumulation of the colouring matter at the circumference. Henle[8] would decide the question by saying that, in a few of these small corpuscles, there are nuclei; but that in the majority (and these the most fully developed,) there are none; so that he thinks it probable that here, as in some other cases, the nucleus, after the cell is perfected, is gradually absorbed.

According to Dr. Barry[9], the young blood-corpuscle in all the vertebrata is a mere disc, with a depression in the centre. In mammalia it retains this form; in the other classes the disc becomes a nucleated cell. The nucleus at first communicates by a pellucid orifice ("nucleolus") with the exterior of the corpuscle, this orifice occupying the place of the depression in the original disc. The orifice becomes narrower, and the nucleus finely granular, and these changes immediately precede the division of the nucleus into minute discs. The discs, whose number is multiplied by successive divisions, and by the gradual appropriation of the nucleus from its circumference towards its centre, arrange themselves so as to form a flat filament, having an appearance the same as that which he finds to be presented by fibre in all the filamentous structures of the body. According to the number of discs, this filament forms within the blood-corpuscle either a ring (as in man and most mammalia, where they are comparatively few), or a coil (as in birds, amphibia, and fishes, where the discs are much more numerous, and the filaments proportionally longer.)

The filament thus formed is flat and deeply grooved on both surfaces, being thereby thinner in the middle than at the edges. The edges are rounded; and,

[1] Lehrbuch, p. 153. [2] Gulliver, in Appendix to Gerber, p. 52.

[3] In the cryptobranchus japonicus (in which there is no persistent branchial apparatus) they measure $\frac{1}{472}$ by $\frac{1}{731}$ (Van der Hoeven, Tijdschrift fur Naturl. Geschiedenis, 1841, p. 270,) but considering the great size ofthe animal, these enormous blood-corpuscles are not disproportionate.

[4] In the blood-corpuscles of the siren, as many as 20 or 30 granules can be seen in one plane of the nucleus. (Owen, in the art. *Siren*, Penny Cyclop.) The proportionally-longest corpuscles are those of the crocodilus lucius: they measure about $\frac{1}{950}$ by $\frac{1}{2538}$ of an inch. (Mandl, Comptes Rendus, Dec. 23, 1839.)

[5] The anarrhicus lupus presents an exception in this respect. Its corpuscles measure $\frac{1}{1750}$ by $\frac{1}{3750}$. (Van der Hoeven, *l. c.*, p. 272.)

[6] Lehrbuch der Phys., i. 154. [7] *L. c.* Appendix, 13.

[8] Allgemeine Anatomie, p. 432.

[9] Philosophical Transactions, 1841, &c. The description in the text has been kindly furnished by Dr. Barry himself. See Appendix A.

when seen on its edge, the filament at first sight seems to consist of segments separated from one another by oblique lines. When perfected, the filament undergoes various changes: sometimes unwinding itself into a straight fibre; at others, continuing circular, while smaller coils of similar filaments are formed within it from a residual portion of the nucleus. In all cases the filament is reproduced by self-division, so that out of a single filament a fasciculus may be formed. Such changes are seen going on in coagulating blood. The filaments now mentioned exactly resemble those which are found in a great many, both animal and vegetable, tissues, nor can any definite line of distinction be drawn among the gradations from them to the double spiral filament, of which Dr. Barry believes that the primitive fibrils of muscle, and certain other tissues, are composed.

In all cases in which a nucleus is present, it differs in chemical characters from the cell. The colouring matter, or hæmatosine, is easily soluble in water, by which it may be completely washed out of the enveloping cells. The latter are composed of a peculiar albuminous substance (*globulin* of Berzelius), which only slowly dissolves in water; the nuclei consist of a different albuminous substance, more like coagulated fibrine, which is quite insoluble in water, and they contain so large a quantity of inorganic matter, that they completely retain their form, and, apparently, their substance, after combustion.[1]

II. Lymph- and chyle-corpuscles. In the villi of the small intestines, the chyle is a pure milk-white albuminous fluid, which does not spontaneously coagulate; its opacity is due to a number of minute oil-globules, varying according to the nature of the food, which float in it without any mixture of fibrine, or of the peculiar corpuscles which afterwards appear. Some of these particles of oil, distinguished by their immeasurably minute size, and by a more general similarity of character than the others present, are described by Mr. Gulliver[2] as forming the *molecular base* of the chyle. Besides these constituents, the more mature chyle, especially after it has passed through lacteal glands, contains the proper *chyle-corpuscles*,[3] which are colourless, moderately transparent, roundish bodies, some larger, some smaller than blood-corpuscles, apparently composed of numerous granules arranged round one or more central molecules. How these are produced, whether by an aggregation of a number of the minutest oil-globules that form the molecular basis,[4] or by a genuine development of cells from the fluid of the chyle, which may be considered as a cytoblastema, is not yet certain; but, be this as it may, as the corpuscles appear and increase, the oil-globules diminish, and the chyle, acquiring a greater proportion of fibrine, becomes more firmly coagulable.

The lymph-corpuscles closely resemble those of the more perfect kind in the chyle. Some of them are generally discernible in the blood, moving, as it circulates in the capillaries, in the peripheral portion of the current. They are somewhat larger than the blood-corpuscles, white, strongly refracting light, roundish and granular, or mulberry-like.[5]

[1] See Harting,(Gissingen betreffende de eerste vorming der cellen, &c., Tijdschrift voor naturl. geschiedenis en physiologie, 1841, 8 Deel;) an essay pointing out several remarkable analogies between the forms assumed by certain inorganic precipitates (such as those of the carbonates of lime and iron) and the forms of the nuclei and cells of organic tissues. The best microscopico-chemical analysis of the blood-corpuscles is in Wagner, (Lehrbuch, p. 160.) The brief notices which can alone be admitted here will but inadequately tell how much aid the microscope has afforded to animal chemistry.

[2] In Gerber, Appendix, p. 88. See also his Contributions, &c. Philosophical Magazine, June 1842.

[3] Discovered by Leeuwenhoeck, and first well described by Hewson.

[4] They do not indeed act similarly on the application of chemical tests; but the observations of Ascherson, presently to be mentioned, show how it is possible that in their aggregation the minute molecules may have their apparent chemical properties modified.

[5] The fullest history of the chyle and lymph is in Nasse, (Unters. zur Physiologie und Pathologie, bd. ii.;) and in Gerber, (Appendix, by Gulliver.) See also Carpenter's Principles of Human Physiology, p. 460.

III. Fat-cells. There are several instances of cells containing oil at certain periods of their development, but those to which the name of fat-cells is peculiarly given, are the minute vesicles which, lying in the areolæ of the fibro-cellular tissue, constitute the adipose tissue. These are true primary cells, whose contents are oil instead of the albuminous fluid with which most are filled.[1] They are, for the most part, nearly spherical, and vary from $\frac{1}{200}$ to $\frac{1}{1300}$ of an inch in diameter:[2] their membranes are structureless and remarkably thin; and they are usually aggregated in bunches traversed and enveloped by fibro-cellular tissue, conveying small blood-vessels.[3] Sometimes each cell bears on its wall a nucleus;[4] and in their early periods the oil in each is not in a single drop, but is composed of one comparatively large and several small drops, which subsequently coalesce.[5]

A fact may be mentioned here by which Ascherson[6] has endeavoured to explain the development, not of the fat-cells alone, but of all primary cells; namely, that if a minute drop of oil be placed in contact with a solution of albumen it directly becomes coated with a film of the latter, so as to resemble a fat-cell. Any one may observe the fact in preparations in which a greasy bone is put in weak spirit: for wherever the oil oozes slowly the bone becomes covered with this artificial fat, for which the filmy envelopes are furnished by the albumen dissolved in the water of the diluted alcohol. It is not probable that the fact admits of an extensive application in the physiology of cell-development: yet there is sufficient analogy between these artificial products and such bodies as the minute corpuscles of the chyle, the granulated oil-globules (*corps granuleux*,) of the milk, and some others, to render it probable that their developmental process is in great measure the same.[7]

IV. Cuticle. The physiology of cuticle has received an altogether new aspect from recent investigations, and especially from those of Henle.[8] He has shown that, with very few exceptions, all the free surfaces of the body, both those of the integuments, of the serous cavities, of the mucous tracts, of the blood-vessels, and of the gland-ducts are invested by a membrane composed of one or more layers of primary cells, forming a cuticle or epithelium.

*Forms and arrangements.* The elements of these cuticles, however, have not all the same form; and while they all serve the common purpose of protecting the tissues beneath them, many, perhaps all, add special functions to which they

[1] Malpighi (De Omento, &c.) first described the adipose tissue with some accuracy. Fontana (Traité sur le Venin de la Vipere, &c.) first clearly described its elementary cells: to Raspail (Nouv. Syst. de Chimie organique) and Schwann (Mikroskopische Untersuchungen), however, are due nearly all the more important facts regarding their minute structure.

[2] See E. H. Weber (Hildebrandt's Anatomie, bd. i.), and Krause (Anatomie des Menschen, bd. i.)

[3] These are admirably figured by Mascagni, (Prodromo della grande Anatomia.)

[4] Schwann, (Mikrosk. Untersuch. über die Uebereinstimmung, &c. p. 144); Bruns, (Allgemeine Anatomie, p. 32.)

[5] Schwann, (*l. c.*) Henle, (Allg. Anat., p. 181.)

[6] Ueber den Physiol. Nutzen der Fettstoffe, Muller's Archiv, 1840.

[7] The minute anatomy of the secernent glands might find its place here, since it is probable the real agents of secretion are isolated primary cells: but most of the glands are of so complex a structure that it will be desirable to describe first some more of their component parts.

[8] Before these investigations the existence of the greater part of the internal cuticles was argued rather than proved. Leeuwenhoeck (Select Works, by Hoole, vol. ii. p. 126,) first described with some accuracy the scales of the epidermis and of the epithelium of the mouth and vagina. Della Torre and Fontana (Traité sur le Venin, &c., t. i. f. 8-10,) more clearly described the cells and their nuclei in the mucus of the eel; then Raspail, Breschet, and others gave accounts of similar cells in the epidermis; and then the cuticles of many different parts were described by Purkinje, Valentin, and other German anatomists. The existence (not the structure) of the epithelium of the intestines was shown by Lieberkuhn (De fabrica et actione Villorum.) There is a complete history of the observations in this part of structural anatomy in Henle, (Allgem. Anat., p. 259.)

are adapted by a peculiar form or energy of their elements. They are all, in their complete state, formed of nucleated cells, in which, while the cells in different parts present many varieties, the nuclei are generally round or oval, flat and colourless, or reddish;[1] and the latter contain one or two distinct small nucleoli, and others very pale and small, and varying in number. The nuclei measure from $\frac{1}{5755}$ to $\frac{1}{3760}$ of an inch in diameter,[2] and the nucleoli about $\frac{1}{10}$ as much. The cells are usually clear and colourless, but are sometimes beset with minute points.

According to the form of the cells, Henle[3] distinguishes three varieties of epithelium; but they are not separated by strict differences, for whenever a continuous surface bears at different parts two different epithelia, there is a very gradual transition from one to the other.

The first is the *tesselated* or *plaster-epithelium,* which is composed of one or more layers of flat, oval, roundish, or polygonal cells, each about $\frac{1}{1900}$ of an inch in diameter, and containing a nucleus of the same shape, which again contains one or more distinct, and several paler, granules. This is by far the most common form of cuticle: it covers the skin and lines the ducts of the cutaneous glands, the mouth, conjunctiva bulbi, pharynx, œsophagus, numerous gland-ducts, the vagina and cervix uteri, the entrance of the female urethra, the serous and synovial membranes, blood and lymph vessels, and a few other parts; and, as a general rule, has a thickness directly proportioned to the friction and other sources of injury to which it is exposed.

*Cylinder-epithelium,* the second variety, is found from the cardia, along the remainder of the digestive canal, to the anus, in most of the gland-ducts that open on the interior of this tract, and in the greater part of the male genito-urinary apparatus and the gland-ducts connected with it. It is composed of closely-set cells of a somewhat conical, pyramidal, or cylindrical form, about $\frac{1}{1200}$ of an inch long, whose apices are attached to the mucous membrane or to flat epithelium-cells lying on it, and whose bases, which are usually terminated by a truncated plane about $\frac{1}{6000}$ of an inch broad, are free. Each such cell encloses, nearly mid-distance between its base and apex, a flat nucleus with nucleoli.

In the *ciliary epithelium,* which constitutes the third variety, cells like those in the second have several fine, pellucid, blunt ciliæ, about $\frac{1}{50000}$ of an inch long, attached to their free extremities. These, by means of some unknown power, are during life in constant motion, either whirling round their fixed extremities, so that their ends describe circles, or else waving continually backwards and forwards and alternately rising and falling. Examples of this kind are found on every part of the respiratory mucous tract above and behind an imaginary plane drawn from the base of the nasal bones to the anterior maxillary spine, even into the air-cells, in the lachrymal sac and canals, along the Eustachian tube, and on the membrana tympani, on the palpebral conjunctiva, the cerebral ventricles, the commencements of the capsules covering the Malpighian bodies,[4] and in the female-genital organs, from the middle of the cavity of the uterus through the Fallopian tubes, and for a short distance on the peritoneal surface of the latter.

The modes in which the elements of these several cuticles are connected are equally various. In the tesselated epithelium the component cells, when there are several layers, generally lie confusedly one over the other; and those in each layer adhere by their edges with the smallest possible quantity of intercellular tissue, their mutual pressure usually making each cell polygonal. In the cylinder variety they are generally almost in contact at their fixed extremities, while their free portions are immersed in a soft intercellular substance, which fills all the spaces between them, and forms a smooth surface over them.

[1] This colour exists particularly in the nuclei of the youngest and deepest layers, (Henle.) Dr. Barry (*l. c.*) points it out as one among many analogies between the epithelium-cells and the blood-corpuscles.

[2] Henle, Allg. Anat., p. 222.

[3] Ueber die Ausbreitung des Epitheliums, Müller's Archiv, 1838; and Allgemeine Anatomie, p. 220, from which nearly all this account of cuticles is derived.

[4] Bowman, (Cycl. of Anat.—*art.* Mucous Membrane;) who denies, also, the existence of ciliary epithelium in the air-cells.

*Modes of growth.* What has been said of the structure of cuticles plainly indicates the change that must be admitted in their physiology. They are, in the most proper sense of the term, organized; for, besides the peculiar definite form of their elements, each cell has in itself the power by which it is developed, and it depends on the subjacent vascular tissue only, as all other elementary structures do, for the supply of the materials which it may use for its own nutrition. These materials before they attain their ultimate form undergo several changes, which have been best traced in the laminated varieties of the tesselated epithelium, including the epidermis and the thickest of the epithelia of the mucous membranes. In all these, the youngest layers, that is, those which lie next the vascular membrane or *matrix*, seem to consist merely of reddish nuclei, and these formed by an aggregation of granules; the cells, if there be any, being too small to be discerned from the nuclei which they contain. As these are moved from the matrix by the new materials successively deposited beneath them, both the nucleus and the cell, which is soon obviously developed around it, grow larger; but the cell outstrips the nucleus, and at length assumes the usual appearance of an oval, nucleated corpuscle. Then, as they become older and still more distant from the matrix, the nucleus either remains stationary or grows gradually more obscure, while the cell still increases in size, but becomes flat, dry, polyhedral, or quite irregular; and at the last (as one sees daily in the outermost layers of the epidermis,) falls off alone or united with many of its fellows in a shapeless scale. In the same progress also its chemical constitution is altered: at first the cell dissolves easily in acetic acid; at last it assumes the peculiar characters of horn, and is altogether insoluble in that fluid.[1]

In the cylinder epithelium the mode of growth is the same as far as the formation of the cell around the nucleus; but then, instead of retaining nearly the same shape, the cell enlarges more in one direction than in the other; and in the ciliary variety proceeds to the production of vibratory ciliæ from one of its extremities.

*Purposes.* The uses of cuticles are probably, as already mentioned, various. One is to protect subjacent parts, another to render certain surfaces smooth. But many may have more active offices. It will be seen that the elements of the most energetic secretory organs have only the same structure as these have; and it is not improbable, for many reasons, that the epithelium-cells also prepare and carry off with them, when they desquamate, some peculiar fluid. The ciliary cells probably serve to fan onward the fluids and the minute particles in contact with them. The direction in which they act is most commonly, but not constantly, towards the external orifice of the canal on which they are placed; but in truth their special purpose is in many instances (e. g. the cerebral ventricles,) as uncertain as the power by which they act.[2]

v. Pigment-cells. In certain parts there are special organs for the production of colour, namely, in the pigmentum nigrum and uvea of the eye, in the membrane of the ampullæ in the internal ear,[3] in the whole of the epidermis of the negro and other coloured races, and in that of the scrotum, the areolæ of the breasts, the labia and some other parts of light-complexioned persons.[4] In

[1] It hardly need be pointed out that these facts prove the distinction of a rete Malpighii from the epidermis to be artificial.

[2] See, on all that relates to them, Sharpey, (Cyclopædia of Anatomy, art. *Cilia;*) Purkinje and Valentin, (Muller's Archiv, 1835; and B. and F. M. R., vol. i. p. 509); Pappenheim, (*ibid.* 1840, p. 510.)

[3] Wharton Jones, (Cyclopædia of Anatomy, *Organ of Hearing,*) to whom also is due the first accurate description of the pigm. nigrum; See Edinb. M. and S. J. xl. p. 77.

[4] Simon, (Ueber die Structur der Warzen, &c. Muller's Archiv, 1840, p. 151.) The black matter of the lungs and bronchial glands has no special apparatus. That which tinges expectoration seems deposited in ordinary mucus-corpuscles, (Martin Barry, Phil. Trans., 1841, p. 226, Pl. xx., f. 72.) The dark colour of many superficial nævi, of liver spots, and of freckles, is due to cells of pigment like those described in the text, (Simon, *l. c.*)

all these the pigment is composed of minute, dark granules, collected in primary cells. The latter, when they lie loosely, as they do on the iris, are oval or round; but when, as in the pigment of the choroid, where they are most distinct, they are set closely and in an even layer, their mutual pressure makes them almost regularly hexagonal. They are nearly flat, about $\frac{1}{1800}$ of an inch in diameter, and lie in apposition so as to form a beautifully tesselated membrane. Sometimes their edges are in contact, sometimes slightly separated by a pale line. Near the middle of each there is a clear spot, produced by the nucleus of the cell, in which there are usually one or two nucleoli; and around this the pigment-granules are arranged. They are most numerous in its immediate neighbourhood, are less thickly collected at a little distance from it, and at the very margin of the cell there are often none at all. They are collected also only at the posterior surface of the cells on the choroid; the anterior portion of each cell (that is, that part which is turned towards the cornea,) contains the nucleus, and is quite transparent.[1] In the negro's epidermis perfect pigment-cells, like those of the choroid, are found only in the deepest layers, and are most numerous at the bottoms of the fossæ between the papillæ. They do not differ from those already described, except in being smaller and in forming several layers; they sometimes seem to have separate granules, not inclosed in cells, mixed with them.

The pigment-granules are among the minutest structures in the body. They are flat, oval corpuscles, measuring about $\frac{1}{20000}$ of an inch in their longest diameter, and about $\frac{1}{4}$ as much in thickness; so that, according to the direction in which they are seen, they appear flat or linear or like mere points.[2] When set free by the bursting of the cell, they exhibit a very active molecular motion; and Schwann has noticed the same occurrence even while they are inclosed. It is only when aggregated that they give their full dark colour; when isolated they are nearly pellucid, or their borders only appear dark.

vi. Nails. Bruns[3] alone has accurately described the structure of the nail in the adult. It consists of primary cells, almost exactly similar to those of the epidermis, firmly fixed together in several layers. The youngest cells (those which lie at the root and in the deepest layers of the body of the nail) are pellucid, round, elongated, or polygonal, and contain a round, granulated nucleus, with some other granular substance. They measure about $\frac{1}{1000}$ of an inch in their greatest diameter, and their nuclei are about $\frac{1}{10}$ as large. The oldest and most superficial cells are larger and broader, but much thinner and more irregular in their shape. Their nuclei are rarely discernible; between the old and new cells, those which are intermediate in situation, are so in structure also. The striæ and fibres, described by Gurlt,[4] are due, Bruns says, to the section of the cell-membranes; the granules of Tourtual[5] are traces of the nuclei.

The growth of the nail is effected by the constant generation of cells at its root and at its under-surface; and this process is carried on with the energy necessary to repair the constant waste, both at the free extremity and the exposed surface; as the successive layers are pushed forward and towards the surface, each cell becomes larger, drier, and flatter, and more firmly fixed to those around it. The identity of the structure of the young cells of nail and of epidermis, renders the question, whether the epidermis be continued under the nail, one of words only.

vii. Cartilage.[6] *True Cartilage.* The proper substance of true cartilage

[1] Henle, Allgem. Anatomie, p. 281. [2] Ibid. *l. c.*, p. 284.

[3] Lehrbuch, p. 197. Before examination he makes the nail very soft by immersing it in an alkaline ley. His description exactly confirms what Schwann had proved of its development.

[4] Unters. über die hornigen Gebilde, (Müller's Archiv, 1837.)

[5] Hornstoff im Kropfen, p.262. Müller's Archiv, 1840, p. 254.

[6] According to their minute structure the parts commonly called cartilages and fibrois a homogeneous and nearly transparent white basis, in which there are numerous small, round, oval, or flat and elongated, or sometimes angular, cavities (*cartilage corpuscles*), from $\frac{1}{1000}$ to $\frac{1}{3300}$ of an inch in diameter.[1] They usually seem to be mere cavities hollowed out in the basis substance; but, by long boiling, the latter is nearly dissolved, while they remain as distinct corpuscles; and sometimes a lining membrane is discernible in them.[2] They are, therefore, to be regarded as genuine cells, whose walls, during their development, have amalgamated with the intercellular substance.[3] Each such cell encloses one or more round nuclei which contain nucleoli, and sometimes are surrounded by cells, so as to give those first mentioned the character of *parent-cells*, that is, of cells inclosing a second generation of cells. The secondary contained cells vary in number, and have usually the form which mutual pressure gives them as they enlarge. The nuclei are often filled by fine particles of oil, which are at first isolated, but may coalesce, and, when very numerous, may fill their cavities so as to make them look like fat-cells; and, when this takes place, the cavity of the primary cell also usually contains particles of oil. But in all these respects, as well as in the character of the contents of the primary cell, whether they be cells or nuclei, and in the mode in which the contained corpuscles are separated or attached to the parent-cell, the varieties are manifold and as yet unexplained.

The number of the cartilage-corpuscles, and the direction in which they are arranged, are more regular. In the articular cartilages they are most numerous, and smallest in the part most distant from the bone. All of them have their long axes vertical to the surface of the bone, except a thin layer of those next the joint, which are flattened and lie parallel to the articular surface. Hence it is that such a cartilage will crack vertically to near its free surface, and then tears transversely. In all other cartilages, also, most of the corpuscles are set vertically to the surfaces, but there is a superficial layer in which they are flattened and lie parallel to them. In the cartilages of the ribs, the internal corpuscles are arranged in rows which radiate from the axis.

The substance in which the corpuscles are imbedded is usually homogeneous and structureless, but in certain cartilages it gradually assumes a finely fibrous aspect, and ultimately becomes truly fibrous.[4] With these changes two others coincide: namely, the formation of fat in the nuclei of the cells, and the acquirement of the yellowish colour in the place of the pearly blue of young cartilage; and all these appear to be preparatory to ossification, for they are never seen in cartilages in which that change does not take place.[5]

*Fibrous Cartilages.* The fibrous structure of some of the cartilages just described makes the transition to the fibrous cartilages, properly so called. In these, there are corpuscles similar to those in the preceding variety (except that the nuclei most commonly contain oil), but the fibres are much darker and coarser, and form the greater part of the tissue. In different examples the directions of the fibres vary: in some, as the symphysis pubis and intervertecartilages may be thus divided (Henle):—1. True Cartilages: the articular (with some exceptions presently mentioned), the costal, ensiform, nasal, trochlea, and those of the whole respiratory tract (with a few exceptions.) 2. Fibrous Cartilages: the intervertebral ligaments, the synchondroses, the cartilage of the ear, epiglottis and Eustachian tube, the Santorinian and Wrisbergian, the sterno-clavicular interarticular discs, and the cartilages of the inferior maxillary articulation. 3. Ligamentous discs: the interarticular ligaments of the lower jaw, wrist, and knee-joints (except the sharp edges of the latter,) the tarsal cartilages, the glenoid and cotyloid ligaments, and the fibro-cartilages of the sheaths and pulleys for tendons.

[1] Meckauer, (De penitiori cartilaginum structurâ, Breslau, 1836.) For measurements in different specimens, see Bruns, *l.c.*, p. 215.

[2] Bruns, Allg. Anat., p. 215.

[3] See Schwann, (Mikrosk. Untersuch, &c.;) Henle, (Allgem. Anat., p. 794.)

[4] These fibres are not demonstrated by snapping a cartilage asunder: the fibrous appearance of the broken surfaces, and the direction of the grain, are due to the arrangement of the cartilage corpuscles.

[5] Henle, Allgem. Anat., p. 798.

bral ligaments, they are parallel; in others, as the cartilages of the ear and epiglottis, they frequently bend, and seem as if they were matted together.

Between these fibrous cartilages and the ligamentous discs which are usually called fibro-cartilages, there are also intermediate structures: the fibres of the fibrous cartilages are not like those of tendinous tissue; those of the ligamentous discs are. But in some examples of the former (the sterno-clavicular and inferior maxillary interarticular ligaments, for instance), the chief substance is traversed by fasciculi of the fibrils of cellular tissue, and thus the transition is established from them to the ligamentous discs which are composed entirely of that tissue.

VIII. BONE—is composed of a basis of apparently homogeneous substance[1] (a compound of cartilage and earthy matter), in which are densely scattered minute cavities with delicate branched canals. The cavities (or bone-corpuscles[2]) are round or oval, and flattened; they measure from about $\frac{1}{2400}$ to $\frac{1}{1680}$ of an inch in length, from $\frac{1}{7200}$ to $\frac{1}{4000}$ in breadth,[3] and are about $\frac{1}{6}$ as thick as they are long.[4] They have somewhat jagged edges, from all parts of which there proceed fine branching canals (calcigerous canals[5]), which traverse the basis substance, and communicate irregularly with one another. The diameter of each canal, at its largest part, is between $\frac{1}{14000}$ and $\frac{1}{20000}$ of an inch; that of the smaller branches is between $\frac{1}{40000}$ and $\frac{1}{60000}$.[6] Müller[7] and Henle[8] have held that the corpuscles and canals are the chief seats of the bone-earth; but Mr. Smee[9] has rendered it more probable that they contain no solid matter, by showing that they can be filled with Canada balsam, and that, in the bones of those who have been embalmed, they are full of a waxen material; and Bruns[10] has found them quite empty in calcined bones, and perfectly transparent in very thin sections. At most, therefore, the earthy matter is more thickly deposited in the walls of the corpuscles and canals than it is elsewhere.

True bone always presents these elementary structures; its coarser structure is variously arranged in different parts, but always preserves a general character of lamellæ arranged round tubes and cellular spaces. The compact structure consists of osseous laminæ from $\frac{1}{5000}$ to $\frac{1}{3000}$ of an inch thick,[11] firmly united in concentric tubes, or in parallel planes, accordingly as the bone is cylindrical or flat.[12] They are most distinct on the exterior of bones, for there they are rarely interrupted by the canals for blood-vessels, which more internally are interposed in great numbers between them, and send off branches which pass through them. They appear either homogeneous or pellucid, or a little granular (like ground-glass), or sometimes very finely fibrous, like the

[1] An exception will presently be mentioned in which it seems to be fibrous. Dr. Carpenter (Principles of Human Physiology, p. 511,) thinks that it is entirely composed of cells adherent together, and filled with a perfectly homogeneous substance.

[2] They were obscurely seen by Leeuwenhoeck (Select Works, vol. ii. p. 129), and badly figured by Mascagni, (Prodromo, Tav. x., xix.) They were first clearly discerned by Purkinje, and described by his pupil Deutsch (De penitiori structurâ ossium, diss. inaug., Breslau, 1834.) They have since been chiefly illustrated by Müller, Miescher, Schwann, Henle, Smee, and Bruns.

[3] Miescher, (De Inflammatione Ossium, p. 42.) The measurements of Valentin, Krause, Bruns, and others are sufficiently confirmatory.

[4] Henle, Allg. Anat., p. 828.

[5] These also were discovered by Purkinje, and more clearly described by Müller.

[6] The latter from Müller, in Miescher, p. 268; the former from Krause. Both are confirmed.

[7] In Miescher, p. 268.

[8] Allg. Anat., p. 829.

[9] On the Structure of normal and adventitious Bone, (Med. Gazette, Nov. 20, 1840.)

[10] Allgem. Anat., p. 241.

[11] Müller, Deutsch, &c.

[12] The microscope was hardly needed for thus deciding the question whether bones are formed of lamellæ. The evidence of old Gagliardi was conclusive. Certain diseases separate these coarser lamellæ; and after softening in acid some may be dissected from all bones.

fibrous portion of the second class of cartilages;[1] and this last appearance is met with especially in cartilages ossified late in life, as those of the larynx, ribs, &c.

The blood-vessels of the compact structure of bone are conveyed in long cylindrical canals (*Haversian canals*[2]), of which the chief run straight in the spaces between the laminæ. These give off small branches which pass obliquely through one or more laminæ,[3] and form communications between the longer canals in different planes, so as to make up a coarse network of bony tubes permeating the compact structure; they open externally to admit vessels from the periosteum, and internally they merge by a kind of gradual expansion into the cells of the cancellous tissue. They are usually cylindrical, and they vary in diameter from $\frac{1}{200}$ to $\frac{1}{800}$ of an inch,[4] those nearest the surface being three or four times smaller than those near the cancellous tissue. Their walls, which are from $\frac{1}{300}$ to $\frac{1}{400}$ of an inch thick, are formed by from five to fifteen concentric lamellæ[5] of bone: and thus, each Haversian canal, whether its course be longitudinal or transverse, is surrounded, as the whole shaft of each cylindrical bone is, by a series of tubes arranged concentrically about its axis. The bone corpuscles lie thickly scattered and as if compressed between the lamellæ, and are so placed that one of their largest surfaces is turned from, the other towards, the axis of the canal. The calcigerous canals run both between and through the several lamellæ; and, since many of them are directed towards the axis of the Haversian canal, they look like a set of faint striæ radiating from it to the outermost lamellæ. Their apertures are discernible in small dots on each lamella, and, according to Krause[6] and Mr. Smee,[7] on the interior of the Haversian canals, into which they believe that some of them open.

Each Haversian canal contains an artery and a vein, which pass along its axis, surrounded by a very soft fat, to which they distribute minute branches. The vessels of the external canals are derived from the periosteum; those of the internal ones from the vessels of the medullary tissue; and the two sets freely anastomose.

As already said, the canals of the compact tissue gradually merge into the cells of the cancellous; and in both the type of structure is the same; for each cell also contains soft fat in which the minute branches of the medullary artery ramify, and the walls of each are formed by a series of delicate lamellæ, with corpuscles and canals arranged in the manner already described.

IX. Teeth. In no organs have the results of recent microscopic researches[8]

[1] Henle, (Allg. Anat., p. 826.)—Malpighi, (Anat. Plantarum, p. 20, &c.) De Lasône, (Mém. de l'Acad. R. des Sc., 1751, p. 98,) and others described bones as formed of laminæ composed of filaments; but these were artificially-made fibres, altogether different from those which are discernible but not separable.

[2] From Clopton Havers, who described them very fancifully in his Osteologia Nova, in 1729.

[3] They form the little processes which Gagliardi (Anatome Ossium, 1689) described as nails. The accuracy of his account of the structure of bones merits something very different from the ridicule commonly attached to his name: the description of Clopton Havers, which is usually praised, is not nearly so good.

[4] Miescher, &c. $\frac{1}{300}$ to $\frac{1}{500}$. Bowerbank, in Medical Gazette, Nov. 20, 1840.

[5] They are called lamellæ for distinction from the *laminæ*, just described.

[6] Handbuch der Anatomie des Menschen, b. i., p. 71.

[7] Medical Gazette, *l. c.*

[8] The chief discoveries were made coincidently by Purkinje, of Breslau, and Retzius, of Stockholm. The former published his observations in 1835, in the dissertations of Fränkel (De penitiori dentium hum. structurâ), and of Raschkow (Meletemata circa dentium evolutionem); the latter in the Transactions of the Royal Academy of Stockholm, and afterwards in Müller's Archiv, 1837. See B. & F. M. R. viii. 158. Interesting as were the facts they revealed, they are far surpassed in importance by the application which Mr. Owen, in his admirable Odontography, has made of the minute structure of the teeth as evidence in determining the general and specific relations of extant and fossil animals. It must be regretted that more use cannot be made of his work in so limited a report as this.

been so unexpected or so brilliant as in these. They have revealed structures before unknown in each of the three component parts of the tooth.[1]

*Cement, crusta petrosa,* or *bone,* forms the outermost layer of the teeth, and in its minute structure differs in no respect from common osseous tissue.[2] It visibly surrounds the whole fang, being thickest near the apex and on the grooves; and Mr. Nasmyth[3] has shown that it also extends in a very thin layer over the enamel of the crown. The existence of this substance which, if not always vascular, can probably easily become so, explains the possibility of ingrafting teeth upon vascular tissue.[4]

*Enamel,* invests only the crown of the teeth, and is composed of solid prisms or fibres, about $\frac{1}{5600}$ of an inch in thickness, set side by side upright on the ivory. One end of each prism is fixed in a little depression on the rough outer surface of the ivory; the other, which is somewhat larger, is turned towards the masticating surface of the tooth in that direction which is best for resisting external force. The course of the prisms is more or less wavy, their curves being, for the most part, parallel, but sometimes opposed. Most of them reach the surface of the tooth; and where they do not, small complemental prisms fill up, like wedges, the vacancies. In the perfect state the enamel contains a scarcely discernible quantity of animal matter, and its prisms are inseparably consolidated; but in young teeth it is soft, and may be broken up into its elements. In this early state it exhibits portions of a membranous animal substance, consisting of the cells in which each of the fibres was enveloped;[5] for the earthy matter is deposited, as it were, in a set of moulds, formed by the primary cells of the enamel-membrane; and as it accumulates, the cell-membrane is so far removed, that in the perfect tooth no trace of it can be discerned; unless, indeed, as Retzius suggests, the fine close-set transverse striæ which go round the whole or a part of each prism indicate the remains of it.

The *Dentine* or ivory forms the chief mass and body of the tooth. It is composed of a fibrous basis traversed by very fine, branching, cylindrical tubuli,[6] which run in an undulating course from the pulp-cavity, on whose interior they open, towards the adjacent part of the exterior of the tooth. Each tubule in its course outwards makes two or three curves, (*primary curvatures,* Owen,) and is, besides, bent at every part into numberless minute and very close undulations, (*secondary curvatures,* Owen;) but the course of those tubules which are adjacent to each other is as nearly as possible parallel. The chief ramifications of the tubules are dichotomous; but they also frequently give off minute branches, which, again, sending off smaller ones, fill up the spaces between the trunks. At the trunk, each tubule has an average diameter of about $\frac{1}{10000}$ of an inch, and the distance between each two tubules is about equal to the width of three. Towards the outer surface of the ivory, the tubules and their branches are immeasurably fine, and some of them pass from the ivory into the bone, and open into the canals of its corpuscles. Both the walls and cavities of the tubules, as well as the substance between them, are filled by the earthy constituent of the ivory which lies in fine granules. The basis of the intertubular substance is

[1] Malpighi (Anat. Plantarum Idea, p. 19, and Op. posthuma, p. 53,) seems first to have described all the three substances. He calls the bone *materia tartarea,* the enamel *substantia filamentosa,* and the dentine or ivory *subs. ossea.* The bone was subsequently overlooked till the time of Purkinje and Retzius.

[2] Its structure also always coincides with that peculiar to the bone of each species. (Owen, *l. c.* xx.)

[3] Three Memoirs on the Teeth and Epithelium; and Med.-Chir. Trans., vol. xxii.

[4] Owen, Odontography.

[5] Schwann, Ueber die Uebereinstimming, &c., p. 118.

[6] Malpighi is commonly quoted as having noticed the tubular or fibrous structure of the ivory; but whoever will examine the places already referred to will find that he never saw the tubules, and only discovered a coarse fibrous network like that of which he wrongly supposed the bones to consist.

[7] Henle, Allgem. Anat., p. 854.

composed according to Henle,[1] of bundles of flat, pale, granular fibres, whose course is parallel to that of the tubules.

x. HAIR. Most of the hairs consist of two distinct substances: an external, cortical, hard, and fibrous part, and an internal, medullary, granular portion, on which their colour chiefly depends.[2] Moderately magnified, hairs look like empty tubes, but in fine transverse sections no central apertures can be seen.

The *cortical part* of the hair is fibrous. Very delicate longitudinal striæ may be traced on it, becoming more faint as they pass from the root to the tip, and in general invisible at a little distance from the latter. They are traceable through the whole thickness of the cortical substance to the very wall of the medullary portion, and indicate the outlines of the component fibres. The latter are, according to Henle,[3] each about $\frac{1}{4000}$ of an inch in breadth, flat, rigid, and brittle, with dark and rough edges. But they may probably be further split, for, after maceration in hydrochloric acid, Bidder[4] found the diameter of the thickest part of a single fibre to be only $\frac{1}{27000}$ of an inch; and Bruns[5] about $\frac{1}{9000}$; so that probably each of the fibres whose course is marked by the striæ is made up of several smaller ones. In some hairs moreover the fibres appear at certain parts, either irregularly, or at definite distances, enlarged; and thus the whole shaft sometimes assumes a beaded appearance.[6]

Besides these longitudinal striæ, indicating the fibrous structure of its cortical part, the surface of the hair is marked by transverse and oblique, and sometimes apparently spiral, wavy lines arranged in a very close series. Meyer[7] has shown that these are formed by the slightly projecting edges of tiers of minute scales, like those of the epidermis, but much smaller, which, being closely imbricated in whorls one over the other, invest the whole surface of the hair, and form a sheath around its cortical part, extending nearly to its tip. They make the hair look as if it were irregularly *hooped* round; or rather, when the hair is very strong, as if it were a closely-jointed reed.

The *interior medullary portion* of the hair is darker than the exterior and granular. It is composed, for the most part, of very minute globules, like pigment-granules or drops of oil agglomerated in small lumps. Sometimes these form one dark mass, continued along the whole shaft of the hair; but more commonly the mass seems broken up, so that there are intervals of different sizes along the axis of the shaft. These are sometimes filled by a substance like the cortical part, and the medullary matter then seems altogether deficient; but more often they are occupied by a colourless substance, clearer and softer than the exterior fibrous tissue. The diameter of this medullary part, when it is completely formed, is about $\frac{1}{3}$ or $\frac{1}{4}$ of that of the whole shaft; transverse sections of hairs exhibit it like a nucleus, with a clear ring around it; along its walls there are often complete pigment-cells, with clear nuclei and transparent membranes.[8]

At the tip, the hair gradually becomes more and more fine, and usually ends in a rounded point, at and near which neither striæ nor medullary substance can

[1] L. c., p. 856. Mr. Nasmyth considers it to be cellular, (Memoirs.)

[2] The medulla is absent in the fine hair over the general surface of the body, and at the very root and near the tip of all hair.

[3] Ueber die Structur und Bildung der menschlichen Haare, (Froriep's N. Notizen, April, 1840, and Allgem. Anatomie.)

[4] Einige Bemerk. über Entstehung, Bau, und Leben der menschlichen Haare, (Müller's Archiv, 1840, p. 538.)

[5] Allgem. Anatomie, p. 204. [6] Bidder, *l. c.*

[7] Froriep's Notizen, 1841, and in Henle, Allgem. Anat. p. 294. Henle coincides in this view: he had formerly (Fror. Notiz., April, 1840) considered the transverse striæ to be due to bands of a substance resembling the elastic tissue wound round the bundles of fibres forming the shaft; a view which closely agrees with that of Dr. Barry, who believes that the hair has the same general structure as the muscular fibre, &c., and is composed of a fasciculus of flat double-spiral fibres, held together by wider coalesced spirals. Valentin (Repertorium, 1841) entirely confirms Meyer's description.

[8] Meyer, *l. c.*

in general be seen. At the root it rather suddenly enlarges into a funnel-shaped extremity, which Henle has named the *knob* of the hair, and which is about three times as wide as the shaft. Just before the hair begins thus to enlarge, the transverse striæ produced by the outermost layers of imbricated scales, are very distinct and broad; but they suddenly cease to be discernible. At the same part the longitudinal striæ become finer, and seem to diverge. But, in addition to these, the knob is marked by coarse, dark, longitudinal striæ, which look like short, interrupted furrows, but which are produced by small, flat, metamorphosed nuclei, about $\frac{1}{1200}$ of an inch long, and $\frac{1}{18000}$ broad. They are largest at the upper part of the knob, and are often tortuous or connected together by fine filaments; lower down they are broader and oval or spindle-shaped, and lower still they pass into roundish or angular granules, like the nuclei of the rete Malpighii. They lie closely in a firm pellucid substance, and sometimes seem surrounded by cell-membranes, among which, in dark hairs, numerous pigment-granules are scattered.[1]

The *knob* of the hair and the nearest part of the shaft are pretty closely invested with a membrane, for which Henle proposes the name of the *sheath*, and of which some or the whole is pulled out when a hair is plucked from the skin. It is continuous with the epidermis, and may be regarded as the epithelium lining the hair-follicle. It is composed of two layers, of which the outer and thicker is yellowish, granular, and thickly set with superficial nuclei, the inner clear and much thinner, and perforated by numerous round, oval, and elongated apertures, but having no trace of cells or fibres. Below, the two layers are united together, and with the exterior of the knob; above, a small space filled with fatty matter intervenes between them and the exterior of that part of the shaft of the hair which is below the surface of the skin.

Cellular and fibrous tissues, or fibro-cellular tissue.[2] All the tissues which have passed under these names nearly agree in their microscopic structure: their chief anatomical differences depend on the mode on which their elements are aggregated. The common material of which they are composed consists of fine, transparent, undulating, cylindrical filaments, from $\frac{1}{30000}$ to $\frac{1}{10000}$ of an inch in diameter.[3] They are generally collected in fasciculi, from $\frac{1}{3750}$ to $\frac{1}{7500}$ of an inch wide, the filaments in which are connected by a firm, structureless cytoblastema; and the fasciculi either merely adhere together, or, as in the case of tendons and other similar tissues, dense bundles are united by others of more loosely-connected filaments placed in their interstices. In different parts either dense or loose fasciculi, or both, are woven into cords, membranes, &c.

In certain situations the elements of the fasciculi are bound together by small filaments of another kind, which, from their supposed origin, may be named *nucleus-filaments*, and which Henle[4] discovered by immersing fibro-cellular tissue in acetic acid, so as to make its proper fasciculi transparent. The latter are thus seen to be severally, and sometimes collectively, constricted at pretty re-

[1] See also on this subject some remarks by Mr. Busk, in the Microscopic Journal, vol. i. p. 26, and vol. ii.; and Simon, Müller's Archiv, 1841, and B. & F. M. R. xiii. 525.

[2] This compound term seems preferable to either of its components. *Fibrous* and *cellular* are both terms applicable to many tissues besides this; but *fibro-cellular* may well indicate a tissue composed of fibres which are in some cases woven so as to inclose cellular spaces.

[3] Treviranus (Beiträge, bd. i.), Jordan (Ueber das Gewebe der Dartos, Müll. Arch. 1834), Gerber (Allg. Anat., p. 123), Henle (Allg. Anat., p. 348), and many others nearly agree in these measurements. Lauth (Nouv. Manuel de l'Anatomiste, &c.) and Krause (Handb. der Anat., bd. i.), make them much greater.

[4] The best tissue for demonstrating this is that which forms the fine fibres connecting the nerves and vessels at the base of the brain; but a similar arrangement exists in nearly every variety. See on that in the pia mater of the spinal cord Purkinje and Luening, (Valentin's Repert. 1841.)

gular distances by one or more dark filaments, very similar to those of elastic tissue, which either wind spirally round them, or encircle them with distinct rings. This arrangement is most frequent when the fasciculi form cords; in other cases (for instance, where many fasciculi lie parallel, as in tendons and strong membranes,) similar dark filaments run, not spirally, but straight along the interspaces between each two fasciculi; and in others (in which the tissue is very lax,) the dark filaments are very numerous and run tortuously, or are even twisted up into little balls.

Other forms discerned in fibro-cellular tissue thus treated with acetic acid, are those of oval corpuscles, like dark cytoblasts, and of elongated, curved, or tortuous striæ, drawn out at their ends. The corpuscles commonly lie in rows with their axes parallel to the proper fasciculi of the tissue; and very often they are connected by longer and more slender bodies, so as to form continuous, undulated, and spiral filaments. Henle, therefore, (to whom all the account of these fibres is due,) has little doubt that the elongated nuclei represent the early state of the dark, *spiral*, and *interstitial nucleus-filaments;* and that, on the other hand, there is a regular gradation from the latter to the filaments of genuine elastic tissue; for all the transitional forms are found mixed with the proper fasciculi in different varieties of fibro-cellular tissue. Again, there is in another direction a transition from the fibro-cellular to the muscular tissue: for though most of the organs composed of the former seem to serve passively their purpose in the economy, yet some, though not distinguished by their structure, have more active properties and contract under appropriate stimuli. Such are the fibro-cellular tissues of the skin and of the dartos, which in their minute structure exactly resemble, on the one hand, the ordinary connecting fibro-cellular tissue, and on the other, the iris[1] and the longitudinal and circular contractile coats of veins and lymphatics, from which there is an easy gradation, both in function and structure, to the genuine organic muscles.

XII. Elastic tissue. (*Tissu jaune élastique.*) In the preceding section the lower forms of this tissue, of which individual fibres are connected with fasciculi of fibro-cellular tissue, are described; in the higher forms of it its peculiar fibres compose independent fasciculi, with which subordinate quantities of fibro-cellular tissue are mixed.[2]

The fibres of elastic tissue resemble, in general, flat, solid[3] bands, of various sizes, from $\frac{1}{4700}$ to $\frac{1}{22500}$ in width.[4] They are peculiarly distinguished by their sharp, smooth, dark edges, their frequent appearance of branching, their tendency to form arches or curls when their extremities are free, and their brittleness, so that they easily break into short pieces with abruptly-cut-off extremities. According to the predominant arrangement of their fibres, Henle divides all the examples of elastic tissue into three varieties. The first, of which the type is in the true vocal ligaments, differs only in degree from the interstitial form of the tissue which is combined with the fibro-cellular fasciculi. Its fibres are undulated, narrow, and very rarely branched or anastomosing; they form, however, independent fasciculi, which are connected by small quantities of the

[1] Krause, Handb., b. i. On the dartos, see Bowman, *l. c.*, who says that it contains organic-muscular fibres.

[2] Henle, Allg. Anat. 399. The best previous accounts were those of Eulenberg (De tela elastica, diss. inaug., Berol. 1836), Schwann (Encycl. Worterb. der Med. Wissenschaften, *art.* Gefässe), Räuschel (De arteriarum et venarum structurâ, diss. inaug. 1836), and Lauth (L'Institut, t. ii. 1834), by whom the peculiar minute structure was first discerned.

[3] Purkinje and Räuschel (*l. c.*) believe that they are tubular, having seen a small dark spot, like a section of a canal, on transversely divided portions of the fibres, and a line of points arranged along the middle of their surfaces. The latter observation, which has been often repeated by Valentin (Repertorium, ii.) and others, may also indicate that the fibre, apparently simple, is composed of two interlacing spirals, like the muscular and others in Dr. Barry's system.

[4] Henle, whose measurements are generally nearly confirmed.

fibro-cellular tissue. In the second variety, which includes the ligamenta subflava, (the most perfect of all the examples of the tissue,) the fibres are large and widely arched, and frequently give off branches. In the third, including the elastic coat of the blood-vessels,[1] the fibres are of smaller size than in the second, and very frequently anastomose so as to form, while they for the most part maintain one general direction, a network with meshes of various size.[2]

When the elastic fibres seem to branch it is doubtful whether a single fibre really splits, or a double fibre divides into its two component parts. Valentin,[3] Lauth, and Eulenberg maintain the latter view; Schwann, Bruns,[4] Henle, and most others, the former; and they regard a genuine branching and coalescing as the peculiar characteristics of these fibres. In Dr. Barry's opinion the reticular arrangement of some of the varieties of elastic tissue, whose fibres have the same general characters as those more simply arranged, affords a striking example of the breaking up of double spiral fibres into networks, which would find a close analogy in the formation of the reticular ducts of vegetables.

XIII. MUSCULAR TISSUE. The transition in function, though not in structure, from the passive to the active fibro-cellular tissue, and from the latter to the organic muscular tissue has been already alluded to. Among the tissues of the present class there are similar gradations in regard to their several functions;[5] but by their structure they may be definitely arranged in two main divisions: the first including the muscles of organic life, which (with one exception) consist of simple, smooth filaments; and the second comprising the muscles of animal life and the heart, which consist of compound and apparently striated fibres, or tubes including fibrils.

A. *Muscles of organic life.* No full account of this tissue was given before that by Henle, who investigated it in his search after the properties of arteries.[6] He says that the fibres, in their most perfect form, are flat, from $\frac{1}{4700}$ to $\frac{1}{3100}$ of an inch broad, very clear, granular, and brittle, so that when they break they often have abruptly-rounded or square extremities. Some of them are uniform; a few bear nuclei; the majority are marked along the middle, or, more rarely, along one of the edges, either by a fine, continuous, dark streak, or by short, isolated, dark lines, or by dark points arranged in a row or scattered; and between these three kinds of marks there are such gradations as prove that they

[1] To be more minutely described in another section.

[2] The elastic tissue occurs in distinct fasciculi in several parts besides these mentioned as types of its various arrangements. For instance, it is found in the bands and ligaments of the larynx and respiratory passages, in a layer surrounding the œsophagus, and in one between the muscular and mucous coats near the cardia and the anus, in the fascia lata, and several other fasciæ, where, besides the interstitial and spiral nucleus-filaments, many independent fasciculi exist. In these parts the arrangement is on the plan of the first variety. Similar fasciculi, but usually arranged in networks, are found mixed in the tissue beneath the epithelium of several serous membranes, as the pleura costalis, the peritoneum on the anterior wall of the abdomen and on the fundus of the bladder, the ligaments of the liver, the intestines, &c. And lastly, fasciculi like those of the ligamenta subflava, are mixed with the proper tissue of many parts of the skin. (See Eulenberg, Lauth, Henle, *l.c.*, and Bowman, Cycl. of Anat., *art.* Mucous Membrane.)

[3] Repertorium, bd. ii. &c. [4] Allg. Anat., p. 75.

[5] The several members of this ascending series may be thus arranged:—1. Common connecting fibro-cellular tissue. 2. The tissue of the skin contracting under the influence of cold and mental emotions. 3. The dartos, which contracts more forcibly under the same influences. 4. The contractile coats of arteries and veins. 5. The iris, which contracts when its nerves are directly irritated, and with the quickness of muscle. 6. The lower organic muscles of the gland-ducts, &c.; and the higher of the stomach, urinary bladder, &c. 7. The muscle of the heart. 8. The perfect muscles of animal life. But it will be observed that the progress in structure does not coincide with this arrangement according to function. The tissue of the iris, for instance, is exactly like the lowest fibro-cellular tissue.

[6] Ueber die Contractilitat der Gefässe, (Casper's Wochenschrift, Mai 1840, and B. & F. M. R. vol. x., and Allg. Anat.)

have all the same origin from nuclei. Fibres such as these are collected in divers numbers in fasciculi, upon which the dark lines just mentioned sometimes form, by branches which they give off and receive, a sort of network, and sometimes run tortuously, like the nucleus-fibres of the fibro-cellular tissue.

Fibres of organic muscle, such as are here described, form the proper contractile coats of the digestive canal from the middle of the œsophagus[1] to the external sphincter ani[2], of the urinary bladder, the trachea and bronchi, the ducts of glands, the gall-bladder, the vesiculæ seminales, the pregnant uterus,[3] the arteries and the veins.[4]

B. *Muscles of Animal Life.* The voluntary muscles are composed of fleshy bundles inclosed in coverings of fibro-cellular tissue, by which each is at once connected with, and isolated from, those adjacent to it. Each bundle is again divided into smaller ones, similarly ensheathed and similarly divisible; and so on, through an uncertain number of gradations, till, just beyond the reach of the unaided eye, one arrives at the *primitive fasciculi*, or the *muscular fibres* peculiarly so called, the first fixed form in the system.[5]

1. *Structure.* The primitive fasciculi consist of tubes of delicate structureless membrane,[6] inclosing a number of filaments. They are cylindriform or prismatic,[7] with five or more sides, according to the manner in which they are compressed by adjacent fasciculi. Their breadth varies in different animals, from $\frac{1}{80}$ to $\frac{1}{1500}$ of an inch; in man from $\frac{1}{200}$ to $\frac{1}{500}$, the average of the majority being about $\frac{1}{400}$.[8] Their most striking, though not constant,[9] characteristics are their pale yellow colour, and their being apparently marked by striæ, which pass transversely round them, in slightly curved or wavy parallel lines, from $\frac{1}{10000}$ to $\frac{1}{12000}$ of an inch[10] apart. Other, but generally more obscure, striæ also pass longitudinally over the tubes, and indicate the size and direction of the filaments, or primitive fibrillæ of which the primitive fasciculus is composed.[11]

The *primitive fibrils* are the proper contractile tissue of the muscle. Each of them is cylindriform, but somewhat flattened, and about $\frac{1}{18000}$ of an inch in its greatest thickness.[12] They are marked by transverse impressions, which are at exactly the same distance apart as the striæ on the surface of the fasciculus.

[1] On the Fibres of the Œsophagus, see the observations of Mr. Gulliver, Proceed. of the Zoological Society, part viii.

[2] In the fibres of the stomach and intestines there is often an obscure division into fine rigid fibrillæ; in those of the upper part of the ureters there is an approximation to the fibro-cellular fasciculi: for they gradually split into undulated fibrils, (Henle.)

[3] Schwann (Mikrosk. Unters. p. 167), Lauth (Müll. Arch. 1835, Jahresb. p. 3), and Baly, in Müller's Physiology. On those of the unimpregnated uterus, which resemble the undeveloped muscular fibres of the embryo, see Purkinje and Kasper, Froriep's N. Notiz. Marz 1842.

[4] See further, p. 266.

[5] They were first described by Hooke, in 1678. The account of them became gradually more accurate in the successive descriptions of Leeuwenhoeck, Muys, Fontana, and Prochaska.

[6] Valentin, (Hecker's Annalen, 1835;) Schwann, (Mikrosk. Unters.) It is the sarcolemma of Mr. Bowman, (Philos. Trans. 1840,) whose complete description of it is generally confirmed by Henle, (Allg. Anat. p. 579.) The latter, however, adds that it is often absent; and that, when present, it bears numerous elongated nuclei like those on the fasciculi of organic muscle.

[7] Muys, (Musculorum Artificiosa Fabrica, pl. i.;) Prochaska, (De Carne Musculari, p. 45;) Bowman, (*l. c.* Pl. xvi. fig. 1, &c.)

[8] Bowman, (*l. c.*, p. 460.) These measurements are generally confirmed by those of Raspail, Schwann, Skey, Henle, and others.

[9] See Gulliver in Gerber, p. 235. See also Martin Barry, (Proceedings of the Royal Society, Jan. 6, 1842.)

[10] Schwann, in Müller's Physiologie, bd. ii. p. 33. Nearly confirmed by Prevost and Dumas, Lauth, Bowman, and others.

[11] Exceptions to these general characters are met with sometimes, but are not yet explained. See especially Gulliver, (*l. c.*,) and Henle, (Allg. Anat., p. 580.)

[12] The measurements of Henle, Lauth, Ficinus, Bruns, and several others, pretty nearly agree with this statement.

Hence it is generally concluded that, as Fontana believed, the striated appearance of the primitive fasciculi is produced by the filaments being so apposed that the transverse marks on all those near the surface lie at exactly the same levels.[1] At present there is much question of the true structure of the fibrils, and of the source of their seeming constrictions, or transverse impressions. Some, indeed, have denied the existence of such constrictions, except when the muscle is contracted, or in some particular condition after death;[2] but the majority differ only in their explanation of them. Some believe that the fibrils are rows of corpuscles, or discs, connected by a homogeneous transparent substance;[3] and others, following Dr. Barry,[4] regard the fibrils as a peculiar form of his grooved and compound filament; each being composed of two spiral threads, wound in opposite directions, and interlacing. In the former view the transverse marks on the fibrils, and the ordinary striæ on the fasciculus correspond to the spaces between the discs; in the latter to the spaces between each two successive turns of one of the spiral threads; for since each filament has its edge turned outwards, only one set of coils can at first be seen.

Each primitive fasciculus contains several hundred of the fibrils; and when fully formed they fill all the cavity of the sarcolemma with the exception of very small interspaces, which seem occupied by a glutinous pellucid fluid.[5] It is only in immature fasciculi that there is an appearance of a central cavity, which is filled either by fluid or by minute granules.[6]

Where a muscle is affixed to a tendon, each primitive fasciculus of the former terminates in an abruptly rounded extremity, which is embraced by a fasciculus of tendinous fibrils, expanding and inclosing it as in a sheath.[7] The coarser fasciculi of tendinous filaments are also continuous with the fibro-cellular tissue which intervenes between the secondary fasciculi of the muscle.

2. *Action.* The actual phenomena of muscular contraction have been often examined in both the voluntary and the involuntary muscles, but the mode in which it is effected is still disputed. Hales,[8] and after him Prevost and Dumas,[9] described the contraction as the result of the primitive fasciculi being thrown into zig-zag lines. But the view of others, (which has been especially illustrated by Mr. Bowman[10]), is that the change is effected by an approximation of the constituent parts of the fibrils (whether discs or coils) which, at the instant of contraction, without any alteration in their general direction, become closer, flatter, and wider; a condition which is rendered evident by the approximation of the transverse striæ seen on the surface of the fasciculus, and by its increased breadth and thickness. The appearance of zigzag lines is referred by Dr. Allen Thomson and Mr. Bowman, to the relaxation of a fibre which has been recently contracted, and is not at once stretched again by some antagonist fibre, or whose extremities are kept close together by the contractions of other fibres.

[1] Dr. Barry, however, (Proceed. of Roy. Soc. Jan. 6, 1842,) says there are states in which the fibrillæ have no share in the appearance of transverse striæ, and in which they are due to the flat and grooved filaments, which, he believes, are wound spirally and interlaced around the bundles of fibrillæ. Some of Henle's observations are strongly confirmatory of this view (Allg. Anat. p. 583); and perhaps Mandl's account (Anat. Microsc. p. 14,) is drawn from a similar appearance.

[2] Treviranus, (Beiträge, bd. ii.;) Ficinus (De Fibræ muscularis forma et structura;) Valentin, (De Functionibus Nervorum;) Skey, (Phil. Trans. 1837.)

[3] For examples (each, however, with some modification,) Lauth, Krause, Turpin, Schwann, Müller, Bowman, Bruns, and Gerber.

[4] *L. c.*, and Philosophical Magazine, April, 1842. Henle, (Allgem. Anat. p. 582,) confesses it impossible to decide the question, but he seems to think that the fibrils are not composed of rows of globules, but derive that appearance from being finely wrinkled. Fontana, also, (Sur le Venin de la Vipere, t. ii. p. 229,) remarked that the appearance seemed due sometimes to rows of globules, and at others to the mere wrinkling of cylinders.

[5] Skey, Bowman, Henle. [6] Valentin, Skey, Gerber, Henle.

[7] Valentin, Bowman, Bruns, &c. [8] Statical Essays, vol. ii. p. 59.

[9] Mém. sur la Phénomènes qui accomp. . . de la Fibre musculaire. (Magendie's Journal, vol. iii. p. 301.)

[10] Phil. Trans. 1840 and 1841. See also Owen and Allen Thomson, in Hunter's Works, by Palmer, vol. iv. p. 261, note; and Martin Barry, *l. c.*

Valentin[1] adopts a middle course of explanation, believing that the production of inflexions in the fibres depends on the degree of their contraction. Ordinary and moderate muscular contraction, he says, is effected by a vermiculation passing very rapidly over the whole length of the fibre, and in this act, the transverse striæ are approximated; but when the contraction is greater, geniculate (zigzag) inflexions are produced, and become the more acute and close the more violent the contraction. All agree in the account of the contraction commencing either at the extremity or at several intermediate parts of a fasciculus, and thence travelling over its whole length; so that the entire act is rather a succession of contractions and dilatations than a single contraction.[2]

In relation to the question of the connexion between the muscular contractility and the nervous influence, the following observation by Valentin may be recorded, but perhaps should not be built upon without much caution.[3] He galvanized on the field of the microscope several minute portions of muscle, and observed whether they contracted or not, which it was possible to do with a moderately high power. Then, with a much higher, he examined whether, in each portion of muscle, there were any portion of nervous fibre included; and he found that in every case in which nerve was present contraction took place, but that when the portion of muscle contained no nervous fibre, the galvanism was ineffectual to produce the same result.

xiv. Nerve.[4]—a. *Structure.* 1. *Cerebro-spinal nervous fibres.* In a nerve belonging to this system one finds numerous fibres inclosed in a common membranous sheath, or *neurilemma.* The latter is composed of fasciculi of fibro-cellular tissue, closely woven together in a generally longitudinal direction, and somewhat more wavy than the filaments in most varieties of this tissue; so that they give the nerve a satin-like glistening aspect. Commonly, also, it has a characteristic striated appearance, (the striæ being transverse or oblique, or even spiral), from a close alternation of bright and dark streaks, which are due to the fibres of the neurilemma being wrinkled,[5] or to the primitive nervous fibres within it being arranged in a slightly tortuous manner,[6] and which are destroyed by stretching and by maceration.

Within this common sheath the nervous fibres are assorted in subordinate bundles of nearly equal size, each of which is inclosed in a separate secondary sheath, continued from the external one, but composed of much finer and less perfectly developed tissue. Within each of these there are again subordinate divisions of the fibres similarly enclosed, till at last, arriving at the primitive fibres, each of these is invested by a covering analogous to the outer neurilemma, but possessing a fineness of structure proportioned to the minuteness of that which it envelopes. It is a most delicate membrane, exactly pellucid, and, in general, seems structureless; but Schwann[7] believes he has seen elongated nuclei in it, and Valentin[8] says he can, in favorable circumstances,

[1] De Functionibus Nervorum, p. 132. His observations were chiefly made by galvanizing portions of muscle on the field of the microscope.

[2] On the application of this to ruptures of muscles, see Mr. Bowman's Paper, in the Phil. Trans. 1841.

[3] And the more since it has not been confirmed, and Mr. Bowman says that in his experiments fibrils contracted after, as he believed, all adjacent tissues were completely removed. It is possible, however, that these were such spurious contractions as, according to Valentin, are produced merely by the action of water.

[4] A better example of the progress of minute anatomy in four years cannot be found than in a comparison of the following account with that in a review, in Vol. VI. of this Journal, of what was known on the same subject in 1838.

[5] Prevost and Dumas, Valentin (Ueber den Verlauf und die letzten enden der Nerven. Nov. Act. Acad. Nat. Cur. 1836, p. 66.)

[6] Fontana, (sur le Venin, &c.;) Burdach, (Beitr. zur Mikrosk. Anat. der Nerven, Ann. des Sc. Nat. 1837, p. 117;) Henle, (Allg. Anat. p. 616.)

[7] Mikrosk. Untersuch.

[8] In Sömmering (vom Baue des mensch. Körpers, bd. v. p. 5.)

see that it has a fibrous appearance, "as if two sets of fibres crossing one another ran screw-like round the nervous tube;" a view which remarkably confirms the observations of Dr. Martin Barry.[1] A longitudinal arrangement of fibres is also sometimes discernible.[2] The proper substance of the nervous fibre, which is contained within the sheath just described, is, according to Valentin,[3] a clear, opalescent, viscous, homogeneous, oil-like substance, without any trace of corpuscles, vesicles, or fibres. Henle,[4] confirming, on the whole, this account, describes each nervous fibre as resembling, in the living or just-dead state, a fine cylinder of clear glass, being pellucid and colourless, and having simple, well-defined, dark edges. But they agree that it is only immediately after removal that this simplicity of composition can be discerned; for very quickly, and especially when they are immersed in water, the fibres, as if by a coagulation of their substance, assume the appearance of being composed of two different materials.[5] In this state their edges are marked by dark parallel outlines, within which again are two internal lines, parallel to them and to one another, which make each fibre look like a tube.

Regarding this last as their natural condition, Remak,[6] whose description agrees very nearly with that of Fontana,[7] considers each fibre as a delicate contractile tube, containing a pale flattened band (the *primitive band*) which, he thinks, is composed of several very fine solid filaments. Rosenthal,[8] also, whose description was written under the guidance of Purkinje, regards each fibre not as homogeneous, but as composed of an *axis-cylinder*, of moderately firm nervous matter, inclosed in a cortical portion of softer substance (*vagina medullaris*); and nearly the same view is entertained by Hannover[9] and by Schwann. Dr. Martin Barry, on the other hand, believes that the nervous fibre possesses a structure analogous to that of the primitive fasciculus of muscular fibrils; that the outer white substance (*vagina medullaris*) is a fasciculus of double spiral flat filaments, and the central portion (*Remak's band*), a filamentous material from which they are continually being given off.

Whatever be the true original structure of the nervous fibre, its cylindrical is soon exchanged for a beaded or varicose form, from the accumulation of the contents into separate masses, which dilate small portions of the sheath while the intermediate spaces collapse; and these changes proceeding, the contents of the sheaths, either spontaneously or by the influence of the fluid in which they are placed, assume a granular or curdy appearance, and may be easily pressed out. From the supposition that these states are natural arose the errors in the first descriptions of minute nervous structure by Ehrenberg[10] and others. The changes take place with the more facility, the more coarsely the fibres are dissected, and the finer and more delicate their investing sheaths are.[11] They are therefore quickly produced in all the fibres of young subjects, and in those

[1] Henle has a less distinct description of a similar arrangement of interlacing spiral fibres around the primitive fibre, (Allg. Anat. p. 620.)

[2] Rosenthal, Valentin. Valentin (Repertorium iii. 262) believes he has sometimes seen movements as if produced by ciliæ on the inner surface of this primary sheath; but he has not much confidence in his observation, (Sömmering's Anatomie, bd. v. p. 6;) and it has not been confirmed by others.

[3] In Sömmering, p. 5.

[4] Allg. Anat. 617. See also Klencke, Ueber die Primitivnervenfasern.

[5] A fact remarkably confirmatory of this view, and of which the first notice is due to Mr. Clift, is the perfect transparency of the living or just-dead retina; it assumes the gray opacity by which it is generally known in the same time as the change here described is supposed to take place in the nervous fibres.

[6] Obs. Anat. et Micr. de Systematis Nervosæ Structurâ, Froriep's N. Notizen, Juni 1838, and in several other papers. See also B. and F. M. R. Vol. VII. p. 500.

[7] Sur le Venin de la Vipere, &c.

[8] Diss. Inaug. de formatione granulosâ in nervis, &c. in Valentin's Repertorium, 1840, p. 76, &c.

[9] Müller's Archiv, 1840, p. 552.

[10] Beobacht. einer auffallenden . . . Struktur des Seelenorgans, Berlin, 1836.

[11] On the influence of particular agents in producing these changes, see Burdach, *l. c.* Valentin, and Henle who also gives a full account of the evidence on all the controverted subjects.

of the brain and spinal cord, and of the nerves of peculiar sensation at all ages. Hence Ehrenberg was led in his first essay to make a marked distinction between the *varicose fibres* in these nerves, and the cylindrical fibres of others; and although his own later observations, and those of all others, prove that all nervous fibres are alike, in their natural state, cylindrical, yet, as Remak has observed, the degrees of facility with which they severally assume the beaded form may serve to distinguish them as well as if they possessed it during life.

The size of the cerebro-spinal primitive nervous fibres is variable, and the same fibres have not the same diameter through their whole length. They are generally largest at their peripheral extremities and in their course along the nervous trunks, where the majority measure, in round numbers, from $\frac{1}{2000}$ to $\frac{1}{3000}$ of an inch in diameter.[1] They gradually decrease in size as they approach the brain, whether directly or through the medium of the spinal cord; and in the brain itself they continue to grow less as they pass through the medullary to the cortical part, so that in the former they measure (on a similar general average) from $\frac{1}{7000}$ to $\frac{1}{8000}$, and in the latter not more than $\frac{1}{14000}$ of an inch.[2] The fibres of the olfactory and optic, and, in a less degree, those of the auditory nerves, are equally small in every part of their course, and thus resemble, in size as well as in structure, those portions of the other nervous fibres which are continued into the nervous centres. Remak[3] also observes that the primitive fibres for common sensation are smaller, and become more readily varicose than those for motion; and Henle[4] confirms this in general, but adds that there is no distinct line of demarcation between the two sets, for that in all mixed nerves fibres of every gradation of size occur.

2. *Organic or sympathetic nervous fibres.* The general neurilemma is tougher in this than in the preceding class of nerves, and has a layer of circular fibres in addition to the longitudinal ones. The several fibres in a nervous trunk are seldom assorted in secondary or more subordinate fasciculi.[5]

In the nerves of this system there are, besides the fibres supposed to be peculiar to them, many of the same kind as those already described; and the latter, which are always finer than the average of the ordinary nervous fibres, vary in their proportionate number in different parts. In the roots of the sympathetic they do not form more than $\frac{1}{4}$ or $\frac{1}{5}$ of the fasciculus; in most of the nerves proceeding from the ganglia to the viscera they are more numerous; in the main trunks, such as the splanchnic and the cardiac nerves, they greatly predominate; and the ciliary nerves, as well as those of the lachrymal and mammary glands and those which accompany the blood-vessels, are wholly composed of fibres like those of the nerves of common sensation.[6]

The nature of the other and peculiar fibres in the branches of the sympathetic nerves has been much disputed. They are described by Remak,[7] Pappenheim,[8] Müller,[9] and some others as distinguished by their fineness, paleness, and yellowish hue, and by the constant absence of the lateral, dark lines which give the cerebro-spinal nerves the aspect of tubes. Rosenthal and Purkinje[10] consider them to be formed by an axis-cylinder similar to that of an ordinary nerve-fibre, and surrounded by a granular nucleated sheath; so that they differ from cerebro-spinal nerve-fibres only in the absence of the outer medullary layer (*vagina medullaris,*) of the nervous substance. Henle thinks they are a peculiar set of nervous fibres arising from the central organs, put into connexion in the gan-

[1] Wagner and Krause, (Müller's Physiology, by Baly, i. 597;) Nasse, (Müller's Archiv, 1839, p.245.) The measurements of several others agree. For comparison, see Ehrenberg, (*l. c.*) and Wagner, (in Burdach's Physiologie, bd. v.)

[2] Treviranus, (Beiträge, hft. ii.;) Henle, (Allg. Anat.)

[3] Vorlaufige Mittheilungen, (Müller's Archiv, 1836, p. 145,) and in other papers.

[4] Allg. Anat. p. 669. The difference is most perceptible in comparing the fibres of the anterior and posterior roots.

[5] Henle, *l. c.* p. 630. [6] Ibid. *l. c.* p. 631-2.

[7] Neurologische Notizen; Froriep's N. Notizen, Aug. 1837, and elsewhere.

[8] Die specielle Gewebelehre des Gehororgans.

[9] Archiv, 1839. Jahresb. cciv.

[10] De formatione granulosâ, &c., in Valentin, *l. c.*

glia, and destined for the contractile fibro-cellular tissue and the blood-vessels. He calls them *gelatinous* nervous fibres, and describes them as pellucid, flat fibres, between $\frac{1}{5600}$ and $\frac{1}{3750}$ of an inch in breadth, with numerous oval or round nuclei arranged at pretty regular distances on their flat surfaces, and more or less elongated and approaching the characters of other nucleus fibres. Sometimes also, he says, the nerve-fibre is split at its extremity like the fasciculus of fibro-cellular filaments in course of development.

On the other hand, Valentin,[1] who is, on both anatomical and physiological grounds, the chief opponent of the notion of a peculiar set of fibres in the organic nerves, regards those which are so described by others as merely the imperfectly developed filaments of fine, fibro-cellular tissue, which are formed into sheaths for the investment, not of collections of nervous fibres, as in other nerves, but of each nervous fibre separately. These sheaths he believes to be continued over the fibres from ganglion globules presently to be described.

The conflicting views may be probably reconciled according to the explanation in a recent description by Volkmann and Bidder,[2] which has received the confirmation of E. H. Weber. It seems most probable that the fibres described by Remak are those of the investing membrane of the true sympathetic fibres, as Valentin holds, and which, in old frogs, exactly resemble the common wavy filaments of fibro-cellular tissue. (E. H. Weber.) But the true sympathetic fibres are still distinct from the cerebro-spinal, and were probably well-discerned by Rosenthal and Purkinje. According to Volkmann and Bidder, they are distinguished by their fineness, (their diameter being constantly about half as great as that of the cerebro-spinal nerves,) their paleness, the absence under all circumstances of a double contour, the very small quantity of curd-like contents which they exhibit when decomposed, and their yellowish-gray colour when they lie in bundles. The difference in size, they add, is so distinct that though both sets of fibres vary to some extent, there is not nearly a complete series of gradations between them.

B. *Course.* The observations of Fontana and of Prevost and Dumas, confirmed by those of Müller[3] and others, prove that the nervous fibre is uninterrupted from its central to its peripheral extremity, and that in all that course there is no anastomosis or confusion of substance between any two primitive fibres—facts proved by both experiment and sight. They have received additional confirmation from Kronenberg[4] and Valentin,[5] the latter of whom examined particularly the nervous fibres in the recti and other minute muscles of very small animals.

The microscope has also materially assisted Volkmann[6] in proving certain facts respecting the course of nervous fibres which could not have been discerned by ordinary dissection. Such, for instance, is the fact, that in several instances fibres proceed for a certain distance from the centres and then, without passing into the substance of any organ, form loops and return again to the brain or spinal cord; so that different parts of the nervous centres may be supposed to be connected by long, nervous fibres, arranged in arches, of which the extremities are at the centres, and the arcs far external to them. This is the case in the plexus of the descendens noni with the cervical nerves, through which some branches of the latter ascend to the brain; and probably also in the arched fibres which form the inner border of each optic tract, and the posterior border of the chiasma; and in those fibres which experiment has nearly proved to proceed in arches from the posterior to the anterior columns of the cord, through the roots of the nerves.[7] It has thus also been made not improbable that the

[1] Ueber die Scheiden der Ganglienkugeln, &c. (Müller's Arch. 1839,) and elsewhere.

[2] Verhaltniss des nervus sympathicus zu dem übrigen nervensysteme beim Frosche, &c. in Froriep's N. Notizen, Marz 1842.

[3] Physiologie, bd. ii.

[4] De plexuum nervorum structurâ, Berol. 1836, and Müll. Arch. 1837, Jahresb. ii.

[5] Ueber den Verlauf. &c. p. 77.

[6] Beob. und Reflexionen ueber Nervenanastomosen, (Müll. Arch. 1840, p. 510), and B. and F. M. R. Vol. XII. p. 239. See also Appendix, B.

[7] Magendie, (Comptes Rendus, Mai et Juin, 1839;) and Kronenberg, (Vers. ueber die motorische und sensibeln Nervenwurzeln, (Müll. Arch. 1839.)

two retinæ are connected by similar arcs, both extremities of which are peripheral; and perhaps there are some other similar instances.[1]

c. *Modes of Termination.* 1. *Peripheral.* This arched arrangement of the nervous fibres is repeated in the substance of the organs in which they are said to terminate; for, as far as they have been examined, the general, if not the constant, mode of termination is as follows: After repeated divisions into smaller bundles of fibres, the fasciculi, which consist of from two to six fibres each, form plexuses, whose arrangement bears a general resemblance to that of the elements of the tissue in which they are placed. These are the *terminal plexuses.*[2] Then each fasciculus of the plexus breaks up into its primitive fibres, and each fibre, either after passing over several elementary structures of the containing tissue, or, as in the sensitive papillæ, the iris, &c., after forming a single narrow loop, returns to the same or an adjoining plexus, and pursues its way back to the nervous centre, from which it set out. In other words, each fibre forms an anastomosis or *terminal loop* with another from the same or a neighbouring fasciculus. There is thus, strictly speaking, no more termination of nerves than of blood-vessels; both alike form circles. The characters of the fibres are scarcely altered in the substance of the organs receiving them: their sheaths become finer, but they are not lost or *laid down*, nor is there any fusion of the nervous into the adjacent substance.[3]

But it is questioned whether this, which is the only really observed arrangement, be a just account of the distribution of the minutest elementary nervous structures. Another view of the whole matter must be taken if it be true that those which are called the primitive fibres be, as the observations of Treviranus,[4] Remak, and Martin Barry indicate, fasciculi, each containing several fibrils which, though they are not discernible in the substance of the tissues, may be distributed to their minutest parts. If it be so, the real ultimate arrangement of the nerves is utterly unknown. There are some imperfect observations respecting the distribution of the minuter fibres, by Schwann[5] and Remak.[6] But it must be admitted as a strong argument against the distribution of such finer branches, that the course of those already described as the ultimate fibres is clearly discernible, that they do not appear to give off branches, and that small as the finer divisions are supposed to be they would not be less than the fibres of many other kinds which can be recognized in their respective tissues.

[1] See Henle, Allg. Anat.

[2] Valentin, Ueber den Verlauf, &c., and in B. and F. M. R. Vol. XI.

[3] No mention is here made of the cells found near the peripheral distribution of the nerves of the higher senses, and supposed by Valentin and some others to be analogous to the ganglion-globules at the nervous centres; for they are not constant elements in the peripheral nervous structures, and Henle's opinion (Allg. Anat., p. 664) that they are epithelium-cells covering the layer of the extremities of the nerves seems more probable than any hitherto offered. Their history may be found in his work, and in the essays on the retina referred to in a following note.

[4] Vermischte Schriften, bd. i. p. 129; and Beiträge, hft. ii. p. 39.

[5] Müller's Physiologie, bd. ii.

[6] Zur Mikrosk. Anatomie der Nerven, &c. In reference to the distribution of the nerves in each organ it must suffice to refer generally to Valentin's edit. of the 5th vol. of Sömmering's Anatomie, and his treatise Ueber den Verlauf und die letzten enden der Nerven; and to Müller's and Carpenter's Physiologies. For particular organs the following references may be sufficient: for the *Retina:* Hannover, (Müller's Archiv, 1840, hft. iii.;) Grube, (ibid. 1840, and Microsc. Journ. 1841, p. 71;) Bidder, (Müll. Arch. 1841;) Remak and Henle, (ibid. 1840;) Gottsche, (ibid. 1834;) Henle, (Allg. Anat.;) and Valentin, (Repertorium, bd. v. &c.) The *Ear:* Wharton Jones, (*Hearing, Organ of*, Cycl. Anat.;) Pappenheim, (Die specielle Gewebelehre des Gehororgans;) Valentin, (Repertorium;) and B. and F. M. R. Vol. VIII. p. 68. The *Nose:* Treviranus, (Beiträge, hft. ii. p. 56.) The *Skin:* Valentin, (Ueber den Verlauf, &c.;) Burdach, (Beiträge, pp. 108, 161;) Breschet, (Recherches sur la Structure de la Peau.) The *Muscles:* Valentin and Burdach, *l. c.* The terminations in the ciliary ligament and iris, the tongue, the blood-vessels, and the teeth-pulps are also described by Valentin and Burdach. The latter shows that in general, though the rule is not universal, the sensitive nerves terminate in plexuses, or complex loops, the motor in simple loops.

2. *Central.* When the nervous fibres have passed centripetally through the dura mater, the general neurilemma becomes thinner, and there is a still further reduction both in it and in the investment of each fibre when they have penetrated the superficial layer of the brain or spinal cord. In the same progress the size of the fibres gradually diminishes, and the tendency to assume the varicose form on the least injury of the investing membrane increases. Having entered the brain, whether directly or through the medium of the spinal cord, the fibres proceed in bundles (which are the coarsely demonstrated *fibres of the brain,*) towards the cortical substance, forming on their way the most intricate plexuses, but never anastomosing. Arrived at the cortical substance, the fasciculi form plexuses among the gray globules exactly comparable with the terminal plexuses already described; and, at the last, the fasciculi, having broken up into their component fibres or very small collections of them, these form loops in the cortical substance of the brain like the terminal loops in the substance of the tissues.[1]

The white substance of the spinal cord, like that of the brain, is composed entirely of the continued nervous fibres. Bundles of them form intricate plexuses without anastomoses, and have all a general direction towards the brain. As far as the microscope can discern, the fibres, as they ascend get nearer to the gray matter, being successively overlaid by those which abut upon the cord higher up than themselves; and the cord, as it passes from below upwards, is thus continually augmented by external layers of fibres, till it comes to the medulla oblongata.[2]

It is not probable that either the brain or the spinal cord contains any fibres but such as are continuations from those of the several nerves of the body: of these they form what Valentin has called a *condensed collection.*

c. *Structure of the Gray Nervous Substance.* The general character of this constituent of the nervous centres is that it is composed of numerous globules, called ganglion-globules, from $\frac{1}{300}$ to $\frac{1}{1250}$ of an inch in diameter, which are usually of a spherical or oval form, more or less flattened, and of a reddish colour. Each contains one or more nuclei with subordinate nucleoli, is inclosed in a very fine filamentous investment, and is often marked with superficial spots of pigment.[3] These investments or sheaths of the ganglion-globules are, according to Valentin and others, formed of several strata, of which the exterior consists of a thin layer of fine granular corpuscles, the next of elongated cells with nuclei, and the most interior and thickest of concentric lamellæ of very delicate cylindrical filaments. By its sheath, each ganglion-globule is isolated from its neighbours; but by the interchange of filaments passing from one sheath to another, the whole of the globules are held together in one group, and lie, as it were, imbedded in the meshes of a network formed by their investments.

The ganglion-globules, which seem sometimes to lie free in their sheaths, so that they may be extracted from them, consist mainly of a red or reddish-gray

[1] Nearly all the minute anatomy of the fibres in the nervous centres is due to Treviranus and Valentin. The arrangement in plexuses has been generally confirmed. It was first minutely described by Dr. Macartney in 1833, in his "Observations on the Structure and Functions of the Nervous System," (see Abstract in Med. Gazette, vol. xiv. p. 842,) The junction of fibres in central loops, first described by Valentin, has been confirmed by Carus, (Einige Aphorismen aus der Phys. des Nervenlebens, Müll. Arch. 1839,) and by Klencke (Ueber die Primitivnervenfasern, Gött. 1841), but by no other anatomist. Valentin himself has lately spoken very doubtfully of it (Repertorium, 1840, p. 96).

[2] The microscope does not confirm the opinion of E. H. Weber, Bellingeri, and Grainger that some fibres pass straightway to the central substance of the cord; nor that, which Valentin's experiments seem to prove, namely, that some of the fibres of the anterior roots which are sent to extensor muscles go at once to the posterior columns, and some of those of the posterior roots to the anterior columns; but the positive results of experiments are in these questions much better evidence than the negative ones of microscopic examination.

[3] The greater part of this account is from Valentin's works.

granular material, held together by a clear soft gelatinous substance; and from this, the ganglia and the gray parts of the brain and spinal cord chiefly derive their characteristic colour. The true original form is probably spherical, but in different situations, either to adapt themselves to the surrounding parts, or according to some law of development, they are elongated to an oval or ovate form, or are heart-shaped, or kidney-shaped, or angular, or altogether irregular. In general, each globule has but one nucleus with one nucleolus in its wall; but numerous exceptions to this also are found which, as well as the varied forms of the globules, seem connected with their progress in development, and with their particular offices in each part.[1]

D. *General arrangement of the two substances in the nervous centres.* The plan of arrangement of the fibres in the brain and spinal cord is already described. The gray substance in the cortical and other parts of the brain, is composed almost entirely of ganglion-globules, which deviate from the general character only in being peculiarly soft, and invested with extremely delicate sheaths. They are collected in small groups in the interstices of the fine vascular network by which the gray matter is everywhere traversed; and the layers, ganglia, and all other forms of gray substance, are due to the different modes in which they are aggregated. On the surface of the cortical substance of the brain, however, another kind of structure is present, and it is found in smaller quantity about other parts of the gray matter: this is a finely granular substance, containing pellucid spherical or oval vesicles, with one or two dark granules in them. In a rather deeper layer, these vesicles, instead of being irregularly scattered through the granular substance, seem each to have appropriated to itself a portion of the latter for an independent covering; and from this condition there seems to be a regular gradation till, in the yet deeper layers of the cortical substance, the vesicles, with their granular coverings, are replaced by perfect ganglion-globules with their filamentous sheaths.[2]

In the purely gray substance of the axis of the spinal cord, the ganglion-globules are arranged in the same manner as in the brain. They are continued even below the giving off of the last spinal nerves in a fine cord-like process, which occupies the termination of the canal of the dura mater.[3]

The ganglia, from whatever part of the nervous system they are taken, present one general plan of structure: their chief mass is composed of a congeries of the ganglion-globules, with which nervous fibres are brought into relation and mingled in various ways. Each ganglionic mass is enveloped by a covering of cellular tissue, continuous with, and analogous to, the neurilemma of the nervous cords that enter into it. The strength of this covering is directly proportionate to that of the sheaths of the globules contained within it; and prolongations from the one are closely interwoven with the filaments of the others.

The nervous fibres that enter into the composition of the ganglia, are similar in structure to those of the sensitive and motor nerves, except that they are finer and their sheaths more delicate. A portion of the fibres which enter one side of a ganglion separate as soon as they have penetrated its substance, and, after forming a plexus in its interior, unite again into one or more cords, and emerge from the other side in the same manner as they entered. But another portion do not pass thus simply: separating from the plexus in individual fibres, or in fasciculi containing each a very few fibres, they wind about in the interior, and usually near the surface of the ganglion, in the most varied manners. In most cases the plexus of fasciculi occupies the central parts of the ganglion, and the fibres and bundles which compose it may be named the *traversing fibres;* the others are, as it were, *spun* round the central parts, and may be called *winding fibres.*[4]

[1] On all these see Valentin, Henle, and Klencke, in the works already quoted.

[2] See Henle, (Allg. Anat. p. 677,) and Valentin.

[3] Remak, Burdach, Arnold, &c.

[4] Valentin, (Ueber den Verlauf, &c.;) Remak, (Froriep's N. Notizen, Aug. 1837.) Nearly all this account of the gray substance is taken from the writings of the former.

The interspaces between the fibres of both kinds are occupied by the ganglion-globules which lie variously entwined by small vessels and nervous fibres. The exact manner of their distribution varies in different ganglia : in some the globules are chiefly arranged around the traversing fibres in the centre of the mass ; in others, they are evenly dispersed throughout, or are absent only at the very exterior ; in others, they are almost all placed at one side. But, however the component parts are arranged, the general plan of construction is in all ganglia the same.

Valentin believes that the winding fibres are those which are about to be distributed in the organs to which they are destined, and that the traversing plexus-forming fibres are those which are as yet far from their destination and which have to pass through other ganglia, in which they also will at last be arranged as winding fibres. There is no appearance of fibres generated within the ganglia; none leave them but such as are continued from those which entered them.[1] Whether they be connected with any fibres of a peculiar structure is already considered : it is only necessary to add, that some of the nerves proceeding from ganglia, and having a gray or reddish-gray colour very plainly marked, contain ganglion-globules mixed with their fibres. This is the case in the connecting cords of the human cervical ganglia of the sympathetic, into all of which ganglion-globules extend from the ganglia themselves,[2] such nerves might indeed be truly called ganglia, for they scarcely differ, except in form, from the bodies usually so named.

XV. Blood-vessels. A. *Arteries.* After a variety of conflicting and unsatisfactory accounts, Henle[3] seems at length to have discerned such structures in the arteries as are adapted to the functions which experiment shows to be performed by them.

His account of their general structure is briefly this: 1st. They have an epithelial lining,[4] consisting of a very thin layer of elliptic or rhombic lamellar cells, which are sometimes elongated into longitudinal spindle-shaped fibres. 2d. There is, immediately external to this, a layer of peculiar tissue, the *striated* or *fenestrated coat*, (corresponding to the *internal coat* of the older anatomists,) consisting of a very thin, rather stiff, and brittle membrane, bearing pale, flat, very narrow fibres, which have, for the most part, a longitudinal direction, and give it a peculiar delicately-striated appearance. This coat, which is often morbidly thickened, and, when an artery is contracted, is commonly thrown into longitudinal folds, is produced by a metamorphosis of the epithelium, which, as the nuclei of its cells disappear, becomes a homogeneous membrane, on which the fibres are afterwards deposited, and which, at last, is completely removed, leaving the fibres free. 3d. In some arteries there is, next, a coat formed by a single layer of longitudinal granular fibres, flat, and tolerably wide, analogous to a coat which is much more prominent in the veins. 4th. A coat composed of *circular fibres* (the *middle* or *elastic* coat of most former writers, the *muscular* coat of Hunter), which forms the chief part of the arterial wall, and comprises all that can be torn from it in a transverse direction. Its fibres are flat, clear, and granular, and break with abrupt ends. Each of them is commonly marked along its middle by dots scattered, or regularly arranged in a longitudinal row, or by a narrow streak: these are the remains of elongated nuclei, which have formed as it were, the pattern, according to which the homogeneous membrane in which they lay has broken up into the fibres.

[1] De functionibus nervorum, p. 66. But see the Appendix, B.

[2] Valentin, (Ueber den Verlauf. tab. vi.) See also, on a similar case in the glosso-pharyngeal nerve, Volkmann, (Ueber die motorische Wirkungen, &c. Müll. Archiv, 1840, p. 488.)

[3] Ueber die Contractilitat der Gefässe, (Casper's Wochenschrift, Mai 28, 1840,) and more fully in his Allg. Anat.

[4] First described by him in his essay, Ueber die Ausbreitung des Epitheliums, (Müller's Archiv, 1838.)

The streaks formed of the elongated nuclei often branch and anastomose, so as to form that kind of network which has led to this coat being mistaken for elastic tissue; whereas it is, in fact, the proper contractile coat of the artery, and is, in all respects of development, and microscopic structure, similar to the layers of organic muscle in the stomach, &c. 5th. On its exterior there is a coat of genuine elastic tissue (*tissu jaune*, the *elastic coat* of Hunter); this exists, however, only in the larger arteries, and its thickness, in comparison with that of the preceding, diminishes in direct proportion to the size of the artery. The direction of its fibres varies greatly in different arteries.[1] 6th. The *external cellular coat* consisting of common cellular tissue, with longitudinal closely-woven filaments.

The conclusions from these facts which, as already said, are the first of the kind that have accorded with the results of experiment and observation of the functions of the arteries, may be expressed in Hunter's words: "From the account we have given of the substances which compose an artery, we may perceive it has two powers, the one elastic and the other muscular. We see also that the larger arteries are principally endowed with the elastic power, and the smaller with the muscular; that the elastic is always gradually diminishing in the smaller, and the muscular increasing, till, at last, probably, the action of an artery is almost wholly muscular; yet I think it is not to be supposed but that some degree of elasticity is continued to the extremity of an artery."

"The muscular power of an artery acts chiefly in a transverse direction;.... the elastic power exists almost entirely in the external coat; the internal coat must be the seat of the muscular power..... Arteries are the conductors and disposers of the blood.....The elastic (power of reaction) is best fitted for sustaining a force applied to it, (such as the motion of the blood given by the heart), and propelling it along the vessel; the muscular power, most probably, is required to assist in continuing that motion, the force of the heart being partly spent, but certainly was intended to dispose of the blood when arrived at its place of destination."[2]

B. *Veins.* The six coats already mentioned include all that are found in the blood-vessels; and the distinctions of the vessels of the several orders depend on the proportional quantities of these coats present in each. The veins, according to Henle, have, 1st, a lining of epithelium, like that of the arteries; 2d, a striated or fenestrated coat, similar to the second in the arteries; 3d, a longitudinally fibrous coat, analogous to that in the arteries, but, in the large veins, formed of several strata, and often morbidly thickened; 4th, a layer, occupying the place of the contractile circular-fibred coat of the arteries, but much thinner than it, and chiefly or entirely composed of fasciculi of cellular tissue, which like that of the skin and dartos may be regarded as contractile; and 5th, the external cellular coat, with longitudinal fasciculi. The true elastic coat is absent.

The valves exist in veins of less than a line in diameter, wherever their office is to be fulfilled.[3] They are covered by the epithelium, and consist of tissue like that of fibrous membranes, which, as Hunter observed, proves that they are not duplicatures of the lining membrane. In the larger valves this tissue is mixed with some like that of the striated membrane of the vein.

Very few conclusions can yet be drawn from these facts, respecting the active functions of the veins. Such as they are, however, they might also be quoted from Hunter, who says, that the veins have nearly the same elasticity with the arteries; that their muscular (contractile) power is very considerable; that the former in some degree preserves them in a middle state; and that the latter adapts them to the various circumstances which require the area to be within that state.

[1] See especially Räuschel, (Diss. inaug. de arteriarum et venarum structurâ. Vratisl. 1838;) and Schwann, (Encyclop. Worterb. der med. Wissench. art. Gefässe.)

[2] Treatise on the Blood, &c. [3] Henle, *l. c.*

c. *Capillaries.* However little the miscroscope may have contributed to the knowledge of the foregoing part of the circulatory system, it has taught all that is known of this, the more important portion of it. It may indeed be regarded as one of its chief honours that it was the means of obtaining the knowledge of the last fact essential to the full proof of the circulation of the blood. Harvey could only prove that the arteries carry blood from the heart, and that the veins bring it back; of the passage from one set of vessels to the other at their distal extremities he knew nothing, and only in the later part of his researches, decided that it was not by the wide channels, which the older writers called *anastomoses,* but probably through a *parenchyma,* in which the blood was infiltrated.[1] The real mode of transit was first proved by Malpighi,[2] in 1661, by a microscopic examination of the circulation in the distended urinary bladder of a frog[3]. His facts were soon confirmed by many others, and especially by Leeuwenhoeck.

*Form and arrangement.* It was not, however, till long after this time that the general existence of capillaries was admitted; and when it was granted, volumes of hypotheses were written about their arrangement, and their various relations to the parts around them.[4] Of late years the microscope has established the truth in far greater simplicity than the imagination had pictured it; proving that, to whatever part the blood is sent, it either passes directly from arteries to veins (both of very small size) or flows from one to the other through a network of minute canals;[5] that it never, at least in the healthy state, passes from the blood-vessels into any other canals or cavities, or into the tissues around them; and that the only mode of communication between the cavity of the vessels, and any other part of the body, is through the invisibly-minute pores, which exist as well in the walls of the capillaries and small vessels as in all organized tissues.[6]

But, though these facts have cleared the way for truth, they have not afforded a deeper insight into the real nature of those processes in which the contents of the capillaries come into immediate relation with the surrounding parts. Marvellous as are the structures revealed by the beautiful art of injection, one cannot yet trace the particular purposes that are served by any of the numerous varieties of vascular arrangement; from Swammerdam,[7] who first employed it as a means of preparation in 1667, to the present day, it has shown increasing wonders of form, but has scarcely afforded a glimpse of the intimate nature of any process.[8]

It will therefore be unnecessary to enter into all the details of the arrangements of the capillaries and small vessels in the several organs and tissues. The

[1] Compare his Exerc. de Motu Cordis, p. 56, and p. 60, (edit. 1766,) with passages in the Epist. Prim. ad J. Riolanum, (p. 105, ej. ed.,) and in the Epist. Secund.

[2] Epist. Secunda ad J. Borellum, (De Pulmonibus, p. 143,) where he describes both capillaries and the circulation in them. The proof by artificial injection seems to have been first obtained by Harvey's friend, George Ent, (Apol. pro circul. sang.) but he could not trace the connexion whose existence he had proved.

[3] The frequent mention of these creatures in physiology will prove how well, in recompense for their sufferings, they deserved the honour of a late essay by M. Dumeril, "Sur les découvertes faites dans les sciences par l'étude de l'organization des grenouilles, (Bull. de l'Acad. de Méd. 1840.)

[4] See especially Haller, (Elementa Physiologiæ, tom. ii.)

[5] Berres calls them, not capillaries, but *intermediate vessels.*

[6] The acute Prochaska was among the first to establish and appreciate the great value of these facts; see his Disq. Anat.-Phys. Organismi Corp. Hum., p. 94, &c.

[7] See his life by Boerhaave, and a communication to the Royal Society in 1672.

[8] The most beautiful delineations of the minute vessels are those by Mascagni, (Prodromo della grande Anatomia;) Berres, (Anat. Microsc. Corp. Hum.;) and Arnold in his recent great work on Anatomy. The differences of arrangement are much less in the capillaries themselves than in the small vessels preceding and following them; it is to these alone that the descriptions which authors give of arborescent, plumose, tufted, and other forms refer.

general facts are these—that the capillaries compose networks permeating the interspaces of the proper elements of each organ and tissue; that the diameters of their canals, (which are all of nearly equal size in the same part,) vary from $\frac{1}{1000}$ to $\frac{1}{5000}$ of an inch, the most common size being about $\frac{1}{3000}$[1]; that the meshes generally bear a close relation in form to the predominant disposition of the proper elements of the tissue, and are in some parts (as the lungs, the choroid, and some mucous membranes,) even narrower than the vessels around them, but more commonly are three or four times wider; and that, as a general rule, the more active the functions of a part, (especially if it be an organ of secretion,) the closer is its network of capillaries.

2. *Structure.* The capillaries have distinct walls, and are not mere channels drilled in the tissues around them. In some parts they seem to constitute the main tissue, as in the pia mater, which is an irregular vascular network with a few cells scattered in its meshes, and the smallest possible quantity of cellular tissue, the vessels in the pulpy membrane of the cochlea of birds, and, as Mr. Bowman has lately discovered,[2] the corpora Malpighiana; in others they are separable from the soft surrounding tissues, as in the choroid, iris, and retina; and in all parts of which the tissues around them are well distinguished by colour and compactness, the walls of the capillaries are plainly discernible. The only question now is concerning the tissues which compose them.

According to Henle,[3] the finest vessels are composed of a completely structureless membrane, in which no fibres or striæ are ever discernible, but which bears minute oval corpuscles, the persistent nuclei of the cells from which the capillaries are formed; they are placed longitudinally upon the vessels, and are arranged in one, or two, or alternate, rows. This may be named the *primary vascular membrane*, because it appears to be the direct product of the primary cell, from which the capillary vessel is formed, and because, in various development, it exists in the vessels of every kind. In vessels of a size just larger than the capillaries, the nuclei of the primary membrane are considerably elongated; and there are added an inner layer of epithelium-cells and an outer layer of pellucid membrane, bearing elongated, transverse cell-nuclei. The latter represents an early stage of the circularly-fibrous coat of the larger arteries. It is from these elongated, longitudinal, and transverse nuclei, that vessels of this size acquire the appearance from which Schwann,[4] and Valentin[5] deduced that they had transverse fibres, and the latter that they possessed both elastic and cellular tissue. Dr. Martin Barry, probably in the same structures, discerns compound double-spiral filaments wound spirally around the vessels.[6]

3. *Functions.* The knowledge of the mode of circulation in the capillaries is entirely due to the microscope, but it must be admitted, that except in Dr. Barry's account of their structure, there is no anatomical confirmation of that which other modes of observation have shown, namely, that they and the small arteries and veins are not merely passive tubes, but may exercise a power of regulating the flow of blood through them.

Under ordinary circumstances the blood moves through the systemic capillaries in an even stream, at an average rate of an inch in a minute and a half, and through the pulmonic system, at the rate of about five inches in the same time.[7] But many circumstances influence the diameter of the capillaries, and

[1] Krause, (Vermischte Beobacht. Mull. Arch. 1837, and B. and F. M. R. Vol. VI.) says there are some much smaller, not more than from $\frac{1}{10000}$ to $\frac{1}{15000}$, and others varying from $\frac{1}{6000}$ to $\frac{1}{9000}$ in the retina, villi, and organic muscle; but his observations have not been generally confirmed. On the whole, however, there is sufficient evidence that in some parts, such as the brain, there are a few vessels not large enough to transmit the blood-globules, (see Henle, *l. c.* 471; and Wagner, Lehrbuch, p. 186.)

[2] On the Malpighian Bodies, &c., Philos. Mag. June, 1842.

[3] Allg. Anat. 491.

[4] Encyclop. Worterb. art. Gefässe.

[5] Repertorium, bd. ii.

[6] Proceedings of the Royal Society, Jan. 6, 1842.

[7] These calculations were made by Hales, (Staticks, vol. ii.) Those by E. H. Weber and his brother, (Müller's Archiv, 1838, p. 450,) make the velocity equal to about 1¼ inch per minute. In either case the result seems inconsistent with the rate at which poisons the motion of the blood in them. Besides the pathological changes which they undergo, the small arteries and capillaries are seen to be contracted by cold,[1] and by warmth to be slightly enlarged; under the influence of certain irritants also, such as capsicum, or an essential oil, they contract, and again, immediately after, dilate,[2] which fully confirms what general observations had made probable: namely, that during life a power is exerted, by which the small vessels, changing their diameter, can control the passage of their contents. And, in like manner, some confirmation has been afforded to the evidence from experiment, that this power is exerted under the influence of the nerves; for an anatomical connexion between the latter and the small vessels has been proved by Purkinje, Valentin,[3] Remak,[4] Henle,[5] and others, who have seen the finest nervous filaments on the wall of the cerebral and other blood-vessels, of less than $\frac{1}{50}$ of an inch in diameter.

M. Poiseuille has greatly added to the knowledge of the current in the capillaries, by watching the *motionless layer* in it. The existence of this layer was observed by Haller, Spallanzani, and others, but its importance was not appreciated by them. From M. Poiseuille's[6] observations, confirmed and extended by E. H. Weber,[7] Gluge,[8] Wagner,[9] and Ascherson,[10] (who has seen the same appearance in mammalia,) it appears that the stream of blood flows most rapidly in the axis of the capillary vessel, and that its velocity gradually diminishes towards the circumference, till, in immediate contact with the walls, there is a layer which is perfectly still. The breadth of this layer, which is the simple result of the adhesion of the blood to the walls of the vessels, is usually from $\frac{1}{8}$ to $\frac{1}{10}$ of that of the whole stream, and is the greater the slower the general current is. Its existence and the different relations of the other parts of the stream are discerned by the observation of the blood-corpuscles. The perfect ones commonly occupy the middle of the stream, surrounded by the lymph-corpuscles, which move ten or more times slower than those in the axis stream. These corpuscles again are surrounded by the motionless layer, into which if any globules are forced, they move very slowly, and, if they come near to the wall, remain for a time quite stationary.[11]

and other substances are proved, by the experiments of Hering and Mr. J. Blake, to be carried with the blood; but the length of capillary tube through which each globule has to pass is extremely small, and in the larger tubes the current is much more rapid. Perhaps also the globules move more slowly than the plasma; if so the conclusions from the microscope must be deceptive.

[1] Schwann and others, confirming Hastings.

[2] See especially Dr. C. J. B. Williams, (Gulstonian Lectures, Med. Gaz. 1841.) Most of the previous observations by Kaltenbrunner, Wedemeyer, Hastings, &c. are nearly valueless, since it is probable that the substances they employed might chemically alter the physical condition of the blood-vessels and the adjacent tissues.

[3] Ueber den Verlauf der Nerven, p. 12.

[4] Obs. anat. et micros. de system. nervosi structurâ.

[5] Allg. Anat. p. 511.

[6] Rech. sur les causes du mouvement du sang dans les vaisseaux capillaires, (Ann. des Sc. Nat. 1836.)

[7] Müller's Archiv, 1837; and B. and F. M. R. Vol. IV.

[8] Sur la couche inerte des vaisseaux capillaires, (Ann. des Sc. Nat. Jan. 1839.)

[9] Nachtrage zur vergl. Phys. des Blutes.

[10] Müller's Archiv, 1837, and B. and F. M. R. Vol. VI. p. 219. Among many deductions which may be drawn from this fact of the blood being at rest near the walls of the vessels, is that the capillaries do not exercise a constant force in propelling it; if they did the part next the wall should move most rapidly.

[11] It is probable that the local influence of cold in making the still layer wider, and retarding the stream of blood, is due as much to some physical influence as to the contraction of the vessels, for M. P. could never discern the latter: he therefore refers the retardation to the well-known rule that the quantity of the same fluid transmitted in a given time by a capillary tube is, within certain limits, directly proportioned to its temperature. He has proved also that the influence of cold is not merely local; but that when it is applied to any part, the capillary circulation is slightly retarded in all; perhaps through the reflex influence of the cold upon the heart.

The fact that the purpose to which the capillaries are habitually subservient is only the passive one of conveying blood close to those parts of the body which either grow or secrete, renders the vascularity or non-vascularity of a tissue a matter of less interest than it used to be; for it is proved that if a part be only able to imbibe the fluid portion of the blood from an adjacent vessel, it nourishes itself as completely, and after the same method, as one whose substance is traversed by numerous capillaries. The extra-vascular tissues, as they are usually called, that is, those in whose substance neither injection nor the microscope has yet revealed any blood-vessels, and which derive their nutritive materials from the blood flowing in adjacent tissues, are the crystalline lens, epidermis, epithelium, and all forms of cuticle, hair, nails, enamel and dentine of teeth, and the analogous structures of feathers, hoofs, &c. To the list of vascular tissues the microscope and the improved art of injecting have added the cornea,[1] the anterior part of the capsule of the lens,[2] the membrane of the aqueous humour,[3] the hyaloid membrane,[4] the articular[5] and other cartilages,[6] the tendons,[7] the elastic tissue,[8] and even the densest bones.

XVI. Lymphatics and Lacteals. 1. *General Structure and Arrangement of the Vessels.* The researches of Panizza,[9] confirming those of Cruickshank, Mascagni, Fohmann, and others, have established that in the tissues generally the lymphatic vessels arise from closely-meshed networks which are interspersed among the proper elements of each part, and which, like those of the capillary blood-vessels, vary in the size both of the canals and of the spaces which they inclose. The difficulty, however, of learning the exact size of these canals is even greater than with the blood-vessels, because of the remarkable yielding of their walls.

There are still several organs and tissues in which no lymphatics have been discovered: such are the brain and spinal cord,[10] the bones,[11] the cartilages, the dense tendons, the eye,[12] the placenta, the umbilical cord,[13] and the membranes of the ovum, and all those into which blood-vessels have not been traced.

The structure of the larger lacteals and lymphatics is very similar to that of the veins. They are lined by an epithelium like that in the blood-vessels; next externally to this is a layer of nearly longitudinal fibres of a character intermediate between those of cellular tissue and the granular fibres of the arterial contractile coat; and around this is a layer of fibres of cellular tissue, which have a circular arrangement, and are connected with those of the next adjacent tissue.[14] The minutest lymphatics seem to be destitute of valves; but they are

[1] Romer, in B. and F. M. R. Vol. II. 235, &c.; Berres, *l.c.*, t. xii. f. 5.

[2] Schroeder van der Kolk, Over choroiditis als oorzaak van Glaucoma, in the Verhand. van het Genootschap . . . te Amsterdam, 1841.

[3] Schroeder van der Kolk, *l. c.*

[4] Berres, (*l. c.* Pl. xiv.;) Dalrymple, (in Tyrrell on Diseases of the Eye.) The most perfect injection is described by S. van der Kolk, *l. c.* See, however, for evidence against these injections, Mr. Toynbee's paper in the Philos. Trans. 1841, p. 159, "Researches tending," &c.

[5] Liston, Medico-Chirurgical Trans. v. 23.

[6] Fremery, Müller's Physiologie, i. 283, &c.

[7] J. P. Medical Gazette, 1839.

[8] Berres, Dœllinger, &c. But this is rather doubtful; the vessels were perhaps in the proper contractile coat of the arteries.

[9] Osservazioni antropo-zootomico-fisiologiche.

[10] Arnold, (Icones Anatom. pars i.) gives admirable figures of the lymphatics of the coverings of these organs; the results of his attempts to inject others in their substance were doubtful.

[11] Cruickshank and Brugmanns believed they had injected lymphatics in bone; but their success is doubtful.

[12] Mascagni (Prodromo) describes some in the eye; but he was too apt to regard every thing as a lymphatic.

[13] See Müller's Physiologie, i. p. 250, on Fohmann's supposed injections.

[14] Henle, Allgem. Anat. p. 551.

discernible in those of less than one third of a line in diameter, and have the same structure as those of the veins.[1] The minutest lacteals in the villi consist of a single membrane with elongated cell-nuclei, corresponding to the longitudinal fibrous membrane of the veins, but not lined by epithelium.[2]

2. *Absorbent Glands.* In the lymphatic and lacteal glands, the walls of the large vessels of which they are mainly composed are traversed by a dense network of capillary blood-vessels; a circumstance which affords some confirmation to the belief that something passes by a kind of secretion from the blood to the lymph and chyle in them, by which the latter becomes more charged with fibrine, and by which the development of the corpuscles is forwarded.

The question whether, in man, the lymphatics and small veins anastomose either in the glands or near their origins, still exists. They certainly anastomose in the latter situation in amphibia and fish,[3] but the microscope has added no evidence to that of Fohmann and the others[4] who hold that they similarly communicate in mammals and birds.

3. *Villi.* The bodies to which this name should be exclusively applied are seated in the small intestines only, and are peculiarly the organs for the absorption of the chyle. They are delicate vascular processes of the mucous membrane, from a quarter of a line to a line and a half in length,[5] of which about twenty-five are set on every square line of surface.[6] They vary in form according as the vessels they contain are empty or full of chyle: in the former case they are flat, and pointed at their summits; in the latter they are cylindrical or clavate.[7] Into the base of each there enters a single lacteal vessel, which, after passing along the middle, ends either in a blind, slightly swollen extremity, or, as Krause[8] and Valentin[9] think, in a network. In some villi, also, there are two such vessels, which pass along opposite borders and terminate without anastomosing.[10] The walls of each are traversed by a very delicate network of blood-vessels formed of from three to five minute arteries, which, after variously dividing and anastomosing, are continued into one or two veins which descend along the villus to the vessels of the submucous tissue.[11] Each villus is farther invested by a very delicate sheath of epithelium, which is frequently, perhaps after each completed digestion, shed.

These facts regarding the structure of the parts of the absorbent system do not at all illustrate their mode of operation. The lacteals have not open orifices in the villi; they probably derive their appearance of terminal apertures from the cells which compose the epithelium over them; they probably, therefore, act by imbibition through their porous walls. But it is to be observed that they do not lie next to the fluid which they absorb; they are covered by a layer of very vascular mucous membrane and by a sheath of epithelium. Valentin[12] has hence been led to suggest that since the blood-vessels in a villus hold nearly the same relation to the lacteal as in a secernent gland they do to the extremity of the duct, and since in absorption the material from the intestines must pass by the blood-vessels to enter the lacteal, it is therefore probable that the chyle is not immediately transferred from the intestine to the lacteal, but is, as it were, secreted into it through the medium of the blood-vessels. Whether this be true or not, the selection of peculiar substances to pass into the lacteals proves that they do not act by so simple a force of imbibition as is exercised by the other porous tissues.

[1] Valentin, Repertorium, 1837. [2] Henle, *l. c.*

[3] See especially Hyrtl. Med. Jahrbucher des Oesterreich. Staates, bd. xxxi.

[4] See Müller, *l. c.* p. 256. [5] Ibid. *l. c.*

[6] Lieberkuhn, Diss. de Fabricâ et Actione Villorum, 1782.

[7] They present many other forms in animals; but these seem to be the only ones that occur in man.

[8] Vermischte Beobachtungen, (Müller's Archiv, 1837.)

[9] Müller's Archiv, 1839. [10] Henle, Symbolæ ad anat. villorum.

[11] Lieberkuhn, (*l. c.*;) Doellinger, (De vasis sanguineis quæ villis ineunt.)

[12] De functione nervorum, &c. p. 142.

Probably the lymphatics of the peripheral network act similarly. Dr. Carpenter[1] has made it highly probable that these do not imbibe all fluids indifferently, but that their office is to absorb the nutritious products of the *secondary digestion;* that sort of digestion which, as Dr. Prout says, is carried on in all parts of the body, and by means of which substances are appropriated for nutrition both from the dead and decomposed elements of all the tissues, and from materials deposited in store for reabsorption, as the fat of hybernating animals.

Experiments have rendered it, on the whole, probable that the coats of the larger lymphatics have vital contractility; and their microscopic structure is favorable to this view. Without such a power the motion of fluid in them is inexplicable; with it, no other is necessary; for the valves, extending as they do into the minutest branches, must render the whole force of the contraction of each segment efficient to the propulsion of fluid into the segment above it.

xvii. Secernent Glands. Many of the general rules of glandular structure laid down by Malpighi[2] and Müller[3] are deduced from microscopic observation, or, at least, they are settled by it, for the objects illustrative of them do not all lie beyond the field of ordinary vision. Of these general rules, the chief are: 1st, That a general unity of plan prevails in the seemingly manifold varieties of glandular structure in the different organs and classes of animals: 2d, That all secretory glands are composed of tubes opening on a free surface, and either simple, or variously ramified so as to present in a small solid space a very great extent of surface for secretion: 3d, That, while the excretory end of the gland-duct opens on a free surface, its opposite or secretory end is always closed: 4th, That aggregations of these blind ends of a ramified gland-duct form the *acini* which were long supposed to be the proper agents of secretion: 5th, That there is no open communication between gland-ducts and other vessels, and that the blood-vessels do not open into the ducts or acini, but ramify in a capillary network in their walls and interspaces, and there supply the materials of the secretion.

Recent observations by Henle, Krause, and others, render it very probable that some of these general rules, though true as far as they go, require to be modified or added to. There are organs which may be strictly called glands, yet have not tubes opening constantly on a free surface, but open thus only at particular times, by a kind of dehiscence. Such are the Peyer's and solitary glands of the small intestines, first well described by Böhm,[4] and since by Krause[5] and Henle;[6] and these may be taken as a type of a numerous class of similar bodies which occur constantly, or at particular times, in the substance of all mucous membranes.[7] Böhm described the Peyer's and solitary bodies as simple sacculi beneath the mucous membrane, without external orifices, containing a fluid rendered opaque by a number of minute white granules and cells, and surrounded by what he called a *corona* of tubules, which had no communication with their cavities. Krause, however, believed he succeeded in injecting some of the sacculi through these tubules; and thus supplied the first step to that which Henle has generalized with other facts of the like kind, in the very probable theory, that all the sacculi of this kind are secretory organs,

[1] Principles of Human Physiology, p. 377.

[2] Epistola de glandularum consimiliumque partium structurâ.

[3] De glandularum structurâ penitiori.

[4] De glandularum intestinalium structurâ penitiori, 1835; see also B. and F. M. R., Vol. I. p. 521.

[5] Vermischte Beobachtungen, (Müller's Archiv, 1837.)

[6] Müller's Archiv, 1833, hft. iv. note; and Allg. Anat.

[7] Lelut, who is quoted by Henle with just praise for his excellent investigations of epithelia, describes numerous glands without ducts in the pharynx and œsophagus, (Des glandes muqueuses, &c. in Journ. Hebdomadaire, 1833, t. 18.) To this class also must be referred the so-called mucous or lenticular glands occasionally met with in the stomach, urinary bladder, &c.

which are closed till the secretion within them is matured, and then, by an absorption or bursting of their finely-membranous walls, open a communication with the surface of the membrane over them, and thus discharge their contents.

If it be admitted that any organ should be regarded as a gland which abstracts materials from the blood, and instead of appropriating them to its own nutrition, discharges them externally, or into some cavity, then, many organs not hitherto regarded as secernent glands may take their place in this class; such as the ovaries, and the so-called vascular glands, which probably elaborate some fluid and add it to the blood in their vessels, or peculiarly alter the blood as it circulates through them. The ovaries, indeed, may be taken as a type of the glands which have not permanently open ducts; for they contain numerous cells, the Graafian vesicles, imbedded in their stroma, which, at the time of conception, bursting through their enveloping membranes, escape into the oviduct, and leave behind them empty sacs. Just so it is also with the Peyer's glands, and all the others of that class; in the place of which we often find fossæ, the remains of the sacculi or vesicles that have recently burst through the mucous membrane, with whose surface their interior thus becomes continuous.

The proper *morphological element* of a gland, then, seems to be a sacculus or vesicle, elaborating within itself or attracting into its cavity the material of secretion, and discharging it through either an occasional or a permanent orifice or duct. These *primary vesicles* (as Henle[1] names them) are probably primary cells more or less metamorphosed. The walls of the smallest among them are formed of a translucent, structureless membrane; those of the largest consist of several layers of elongated nuclei and filaments of cellular tissue, arranged in concentric circles around them, and are sometimes lined by an epithelium. These additions, it is presumed, are effected by the development of nuclei and cells from the primary cell-wall; as in the blood-vessels the epithelium and the striated and fibrous coats are produced from the primary vascular membrane. The structure of the larger permanent gland-ducts also, when any can be separated from the vesicles, is very analogous to that of the blood-vessels, and is perhaps the result of similar phases of development. They consist of an internal layer of epithelium-cells (generally like those of the adjacent membrane), surrounded, first, by a layer of longitudinal fibres similar to those of organic muscle, then by a much thinner, and not always discernible, layer of circular fibres of the same kind, and lastly by a layer of cellular tissue.

According to the mode in which the primary gland-vesicles arrange themselves, three different forms of glands with permanent orifices may, according to Henle,[2] be produced. 1st, The *closed tubular glands*, which are formed of a single elongated vesicle, or in which a number of vesicles may be supposed to have arranged themselves in one line, and then to have all opened into one another by their apposed portions, except the lowest, which has remained closed at one end, and the one nearest the surface which has opened externally. Such are the Lieberkuhnian and tubular glands of the intestines, and the simple tubular glands of the stomach, mere tubes, like single gland-vesicles elongated to different depths, with walls, composed of a tube of structureless membrane, opening permanently on the mucous surface, and closed at their opposite ends. Such also, though rendered more complex by the attachment of vesicles along their sides, are the Meibomian glands; and such, elongated and grown tortuous, are the perspiratory and ceruminous glands. 2d, The *aggregated* glands, in which a number of vesicles arranged in groups have become so connected by a kind of fusion of their adjacent walls, that only a small portion of the membrane of each remains, and they form one cavity, with numerous recesses from its inner circumference. Such are all those commonly called mucous glands (as those of the lips, trachea, vagina, &c.); and the tonsils, lachrymal, Brunnian, salivary, mammary, and Cowper's glands, the pancreas and prostate; which differ only in secondary points of structure, such as the arrangement of their excretory

[1] Allgemeine Anatomie, p. 821, e. s.

ducts, and the mode in which the *primary lobules* or simplest groups of gland-vesicles are connected together by fibro-cellular tissue, and supplied by blood-vessels. The smallest branches of the gland-ducts sometimes run into the central cavity of the group of vesicles, which thus all open into it: sometimes the groups, or primary lobules, are set upon the extremities or by the sides of the ducts: but whatever secondary arrangement there may be, all have the same essential character of rounded groups of gland-vesicles opening by a common central cavity into minute ducts. 3d, The *reticulated* glands, such as the kidneys and testicles, which consist of tubes of a transparent and structureless homogeneous membrane, the *membrana propria,* probably formed by the elongation and anastomosis of cells, like the blood-vessels, or the Haversian canals in bone, and which, like them, are connected in a network, and seldom or never end in a cul-de-sac.

Whether the gland be a vesicle which only once or occasionally opens, or one which, perhaps after sundry metamorphoses, has a permanent communication by ducts with the external surface, the mode of secretion seems to be the same. It is, however, still a question (and one which the microscope will not decide,) whether the fluid separated by the vescicles be always already formed in the blood, or whether it be not in some cases elaborated and transformed in them. Probably there is not one rule for all glands; for in certain secretions the microscope detects constituents which could not have been separated as such from the blood, namely, globules and corpuscles of different kinds, which indicate that the fluid separated had the character rather of a cytoblastema than of a dead matter.

In those secreted fluids which serve especially or solely for the purification of the blood, and are therefore more peculiarly excretions, such as the bile and the urine, there appear to be, during health, no corpuscles. But in those which after their discharge have to serve some special office in the economy it is very usual to find cells either perfect or in various stages of development. Henle has treated at some length of these endogenous cells. At present it may suffice to say of them, that they sometimes constitute the essential part of the secretion, as in the testicles, where they either become or generate within themselves the seminal filaments or animalcules;[2] the ovaries, where they become the germinal vesicles; the mammary glands, where they are the peculiar milk-corpuscles; the gastric glands, where they contain digestive fluid. In other cases they form an epithelium to line the gland-vesicles and tubules: in others, without any evident purpose, they are discharged from the gland-ducts or fill their extremities in the form of mucus-corpuscles; in others they appear as globules or cells of fat.

From the preceding account it is plainly not possible to draw a strict line between secretion and nutrition. In both alike the fluid abstracted from the blood may work in itself changes characteristic of life. Neither can expulsion from the seat of their production be regarded as characteristic of secreted substances; for the elements of many epithelia remain attached only a little longer than the corpuscles of some secretions, and the attachment of some of them is perhaps even shorter, as, for instance, of the epithelium-cells of the stomach in comparison with the animalcule-generating cells of the semen.

The ducts of glands which have permanent external orifices and through which the secretion formed in the gland-vesicles is discharged, have a minute structure very similar to that of blood-vessels. They are lined by a delicate layer of epithelium-cells, continuous with those of the surface on which they open, and continued to their finest branches. The forms of its elements in

[1] The *basement membrane* of Mr. Bowman, (Cycl. of Anat. *art.* Mucous Membrane,) who suspects that a similar membrane also lies beneath all epithelia.

[2] See some observations illustrative of this by M. Lallemand, in the Comptes Rendus 1841; and Edinb. Med. and Surg. Journ., 1841. See also Martin Barry, *l. c.* and Kolliker Ueb. das Wesen der Saamenthiere. Fror. N. Notiz. Juli, 1841.

different glands have been already mentioned. (p. 245.) Outside it there is in most instances a layer of fine, longitudinal fibres exactly resembling those of the contractile coats of the arteries and veins, and of the other lower muscles of organic life; and surrounding this a second thinner layer of similar fibres arranged in circles.[1] Lastly, external to these there is a layer of fine fibro-cellular tissue, but, considering their volume, there is scarcely any class of organs which contain so little of this tissue as the secernent glands.

XVII. VASCULAR GLANDS. Under this name may be classed the thymus and thyroid glands, the renal capsules and the spleen, which, as Henle[2] well says, " agree chiefly in this, that both their minute structure and their physiological import are at present totally unknown." It may suffice, therefore, to refer to the chief works in which there are original descriptions of their minute structure.[3]

XVIII. ERECTILE TISSUE. This might perhaps justly have had its place with the description of the organs of circulation since the only function which it discharges seems to be chiefly due to the peculiar arrangement of its blood-vessels. The parts in which it is found, however, are sufficiently similar in their characters to form a separate class: they are the penis, the clitoris, and, perhaps, the nipples. The first has been so much more examined than the others that the description of its structure is taken as the type of theirs.[4]

Independently of its blood-vessels, the erectile tissue of the penis is composed of a network of cords and bands, which form a multitude of freely-communicating cells, of various shapes. They are chiefly derived from the fibrous envelope of the penis, from which they pass on either side inwards, connecting the septum with the outer sheath. The substance of these cords and bands is traversed by the arteries of the erectile tissue, which usually run very tortuously within them: the spaces or cells between the cords and bands are occupied by the veins. Each cord or band has in its interior an artery of a size proportionate to its own: where one seems to branch, the artery within it also divides, and sends a branch to its division; and where the bands and cords unite, the arteries

[1] The descriptions of the muscular fibres are drawn chiefly from the gland-ducts of large animals; such as the ureters and bile-ducts of sheep and horses. (See especially Meyer, De musculis in ductibus eff. gland., diss. inaug., Berol. 1837; and in Froriep's N. Notizen Marz 1838; and Henle, Allg. Anat. p. 934.) The same structures are presumed to exist in man rather from analogy and from pathological facts than from actual observation. Tourtual has discovered true muscular fibres in an hypertrophied human ureter, (Muskelfasern im erweiterten Harnleiter, &c., Muller's Archiv, 1840;) but there are no detailed accounts of the muscular coats of other human gland-ducts.

[2] Allg. Anat. p. 996.

[3] *On all of them,* Berres, (Anat. Microsc.;) Müller, (Physiologie,bd. i. and Archiv, 1840, p. 101;) Burdach (Physiology, bd. v.;) Henle, (Allg. Anat. p. 996:) and Gulliver, (App. to Gerber's General Anat.) *On the spleen:* Giesker, (Splenologie, Zurich, 1835, quoted by Henle;) Müller, (Archiv, 1834;) Valentin, (Ueber der Verlauf der Blutgef. in dem Penis, &c. Müller's Archiv, 1838;) Bischoff, (Müller's Archiv, 1838;) Marcus, (Diss. de funct. lienis, 1838;) and the earlier works of Hewson, Heusinger, Schmidt, and Meckel. *On the renal capsules:* Nagel, (Ueber die Structur der Nebennieren, Müller's Archiv, 1836;) Bergmann, (Diss. de glandulis suprarenalibus, 1839;) Pappenheim, (Vermischte Beobacht. Muller's Archiv, 1840;) and Berres, (Ueber den zarten Bau der Drusen, Oester. Med. Jahr. 1840.) *On the thyroid and thymus glands:* A. Cooper, (The Anatomy of the Thymus-gland;) Berres, (*l. c.* in Oester. Jahrbuch;) Haugsted, (Thymi in homine, &c. quoted by Henle.)

[4] The only two complete descriptions are those of Müller, (Entdeckung der bei der Erection . . . wirksamen Arterien, Archiv, 1835;) and Valentin, (Ueber den Verlauf der Blutgefässe in dem Penis, Müller's Archiv, 1839,) with a subsequent note by Müller. This account is so far drawn from both equally that they will be referred to only when they differ.

within them also unite and anastomose. The terminations of the arteries open in an uncertain manner from the cords which contain them into the veins which are placed in the interspaces of the cords and bands, and of which the latter, since they are covered by the lining venous membrane, may be said to form the walls.

These bands and cords which form, as it were, the skeleton of the erectile tissues are dense structures, composed of several different tissues. From its surface inwards each consists of the following layers: 1st, a portion of the common lining membrane of the venous system, which is here, as elsewhere, in contact with the venous blood; 2d a mixed layer of elastic and fibro-cellular tissue, which corresponds to the outer wall of the vein or venous space; 3d, a layer of fasciculated fibres, exactly resembling those of organic muscle, and usually directed in the longitudinal axis of the penis; 4th, one of bundles of fibres, like those of tendon; 5th, the artery which occupies the middle of the band or cord, and on the other side of which the same succession of layers is usually repeated, though less regularly, because on one side or other of each band there are generally branches given off.[1]

Thus far there is little question as to the structure of the true erectile tissue: the chief doubt is in regard to the mode in which the arteries pour their blood into the veins. Müller believes[2] that they have two distinct modes of termination; that some of them—the nutritive branches—form a common capillary network, which leads in the usual manner to the minute veins; but that, besides these, there are others, which he names *helicine arteries*. These, he says, form branches about a line long and $\frac{1}{100}$ of an inch thick, which proceed, usually at right angles from the branches of the arteries within the bands and cords, and hang loose, with blunt and curved or twisted, but not open, extremities in the venous spaces. Through these the blood is, in erection, supposed to be poured into the veins, which are thus distended. Valentin,[3] on the other hand, maintains that the appearance of the helicine arteries is artificially produced; that, in cutting across a collection of cellular spaces like those of the corpus cavernosum, many of the bands and cords which bound and traverse them must be divided; and that since each of these contains in its interior a tortuous artery, it must, when cut away from one of its connexions and floated out in water, present the appearance of the end of an artery hanging loosely in a venous space. He believes, therefore, that the minutest branches of the arteries of the penis, after various anastomoses, dilate, penetrate the fibrous tissue in which they are inclosed, and pass into the smallest venous spaces, or (as it may be better expressed, in accordance with Berres' injections,) into a dense network of comparatively large veins, whose diameter is four or five times greater than that of the arteries, and the interspaces between which are formed by the intersecting fibrous bands and cords, in the substance of which the arteries run.

### APPENDIX.

A. An observation which the writer, in common with many others, had overlooked, but which seems to confirm in some measure Dr. Barry's account of the blood-corpuscles, is made by Mayer, of Bonn.[4] He says that he has often found floating in the blood of men, mammalia, and birds numerous pellucid,

[1] This description is drawn from the corp. cavern. of the ass; the same structures exist, but are less discernible in that of man.

[2] And he is, in general, confirmed by Krause, (Vermischte Beobacht, Müll. Archiv, 1837;) Hyrtl, (Oester. Jahr. 1838, bd. xix.;) and Erdl, (Muller's Archiv, 1841.) But see on their observations, Valentin, Repertorium, 1841.

[3] His observations are confirmed by Berres, (Ueber den zarten Bau der Drusen, Oester. Jahrb. bd. xxxi.)

[4] Ueber freie Primitivfasern im Blute. (Froriep's N. Notizen, April, 1841.)

clear, straight, smooth, or somewhat granulated fibres, varying from $\frac{1}{1100}$ to $\frac{1}{550}$ of an inch in length, (but sometimes much longer,) and about $\frac{1}{20000}$ of an inch broad. In a pea-fowl which had died of acute abdominal inflammation they were as numerous as the blood-globules themselves, appeared notched, or granulated, and had independent motion. He believes also that similar fibres exist in several parts of the body: e. g. in the parenchyma of the spleen and kidney.

B. While the Report was being printed, the observations of Volkmann and Bidder, of which a part are inserted, arrived in the March number of Froriep's Notizen. They seem to prove anatomically the important fact, that the sympathetic nervous fibres do form an independent system, whose centres are the ganglia. At the points of junction of the two systems some of the sympathetic fibres (traceable by the characters described at p. 262,) run towards the spinal cord, others towards the periphery. The proportionate numbers passing in each direction vary in the different places of connexion. From the branches connecting the sympathetic with the eighth and ninth spinal nerves of the frog, for instance, scarcely any sympathetic fibres proceed centrally; yet these are the largest of the branches which are commonly described as the *origins* of the sympathetic system from the spinal cord. On the whole, the sympathetic at these points of junction always gives more fibres that it receives. It must therefore have some source of fibres of its own; and this source is found to be chiefly in the ganglia, both spinal and sympathetic, (specially so called,) and, in a less degree, in the cord itself.

If, it is argued, the sympathetic derived at these parts all its fibres from the spinal cord, such fibres ought to be found in duly proportionate number in the roots of the spinal nerves. But it is not so. In the case of the fourth spinal nerve of the frog, for instance, the branch connecting the sympathetic with it, is larger than *it* is. Therefore, since all the fibres of the connecting branch run centrally, they ought, if they have their origin from the spinal cord, to be found in great numbers in the roots; but there are fifty times more cerebro-spinal than sympathetic fibres in the roots of this fourth nerve. The sympathetic fibres which pass into its trunk cannot be traced further than to the ganglion on the posterior root. From this ganglion, therefore, they probably have their origin; and they are destined, as those in the other trunks are, chiefly to the posterior branches of the nerve. The anterior branches also contain sympathetic fibres, but they are derived, not from the spinal ganglia, but from those of the sympathetic itself.

*June* 11, 1842.

